# Cellular responses to beating hydrogels to investigate mechanotransduction

**DOI:** 10.1038/s41467-019-11475-4

**Published:** 2019-09-06

**Authors:** Yashoda Chandorkar, Arturo Castro Nava, Sjören Schweizerhof, Marcel Van Dongen, Tamás Haraszti, Jens Köhler, Hang Zhang, Reinhard Windoffer, Ahmed Mourran, Martin Möller, Laura De Laporte

**Affiliations:** 1 DWI – Leibniz-Institut für Interaktive Materialien e.V, Forckenbeckstr. 50, Aachen, 52074 Germany; 2 Institute of Molecular and Cellular Anatomy, Uniklinik, RWTH Aachen University, Aachen, 52074 Germany; 3 ITMC- Institute of Technical and Macromolecular Chemistry, RWTH Aachen University, Aachen, 52074 Germany

**Keywords:** Cell migration, Cell signalling, Biomaterials, Gels and hydrogels, Polymers

## Abstract

Cells feel the forces exerted on them by the surrounding extracellular matrix (ECM) environment and respond to them. While many cell fate processes are dictated by these forces, which are highly synchronized in space and time, abnormal force transduction is implicated in the progression of many diseases (muscular dystrophy, cancer). However, material platforms that enable transient, cyclic forces in vitro to recreate an in vivo-like scenario remain a challenge. Here, we report a hydrogel system that rapidly beats (actuates) with spatio-temporal control using a near infra-red light trigger. Small, user-defined mechanical forces (~nN) are exerted on cells growing on the hydrogel surface at frequencies up to 10 Hz, revealing insights into the effect of actuation on cell migration and the kinetics of reversible nuclear translocation of the mechanosensor protein myocardin related transcription factor A, depending on the actuation amplitude, duration and frequency.

## Abstract (web summary)

Mechanotransduction of cells is of interest for a number of reasons but model in vitro systems remain a challenge. Here, the authors report on a hydrogel which changes properties upon near infrared irradiation to create cyclic forces and demonstrate the application of these gels to study mechanotransduction.

## Introduction

The extracellular matrix (ECM) is dynamic and undergoes constant remodelling, partly due to forces exerted by the cells. Therefore, the ECM provides changing mechanical cues to the periphery of the cells, which are again conveyed by the cells to their internal machinery affecting cell behaviour. This communication process is called mechanotransduction and takes place in a continuous feedback cycle. A disruption in force transmission is associated with many pathologies and diseases, ranging from arteriosclerosis, muscular dystrophy and cancer^1,2^. Importantly, many soft tissues, such as the heart, blood vessels^3^, lungs^4^ and cartilage^5^ often experience cyclic, rather than constant strains. Deciphering this language of forces is, however, hindered due to the lack of in vitro systems that can generate and regulate forces on cells that mimic natural stresses. For example, low throughput cell-selective manipulation techniques that target individual cells, including optical tweezers, atomic force microscopy, and magnetic bead cytometry^6,7^, resemble acute, invasive stresses (cell poking), which may damage the cells by affecting their cortical shell^8,9^. On the other hand, more realistic stresses can be applied by a growth substrate via stretching devices (e.g. Flexcell, USA). Here, cells are grown on flat or patterned stiff polydimethylsiloxane (PDMS) elastomer sheets (elastic modulus (E) ~2 MPa) and exposed to oscillating strains consisting of amplitudes ranging from 0 to 23% and frequencies as high as 5 Hz (higher frequencies limit achievable maximal strains)^10–12^. A majority of systems have a large response time (~s) and are restricted to generate unidirectional or bi-directional forces without spatial control of the region, in which cells are affected.

To manipulate cells with precisely controlled mechanical forces in space and time, one other approach grows cells on cell-adhesive stiff elastomeric micropillars (Norland Optical Adhesive (NOA), E ~1 GPa) that are embedded in a thermoresponsive, non-cell-adhesive poly N-isopropyl acrylamide (PNIPAM) hydrogel containing gold nanorods (AuNRs) as photothermal transducers. Light triggered local change of the hydrogel results in bending of the elastomeric micropillars^13^, which substitutes the electrical motors used in traditional cell stretching devices. In contrast to natural substrates, where cells are free to grow and attach, here cells are restricted to attach to the micropillars, which limits their spreading and migration behaviour. This technique uses relative low frequencies up to 0.1 Hz and does not stimulate cells for long periods of time, hence no link with mechanical cell signalling pathways was revealed. To better mimic the ECM and grow cells on 2D^14^ or in 3D^15–18^, soft hydrogels (E ~0.1–100 kPa) are often used. While most traditional elastic, flat and static hydrogels do not represent the dynamic nature of native ECM, some engineered hydrogel materials enable spatio-temporal changes in a unidirectional manner using external triggers^18^, such as light-induced softening^19,20^ or stiffening^21^ that cannot be reversed. Only a few hydrogel systems offer dynamic, reversible changes in mechanical properties without spatial control^22–24^ or undergo slow transitions (in the order of days^25^, hours^24^ or minutes^26–28^), while others are not compatible with cells^29^. Currently, to the best of our knowledge, no soft hydrogel systems are available that facilitate both cell adherence and reversible, cyclic mechanical property changes up to 10 Hz with precise spatial control.

**Fig. 1:**
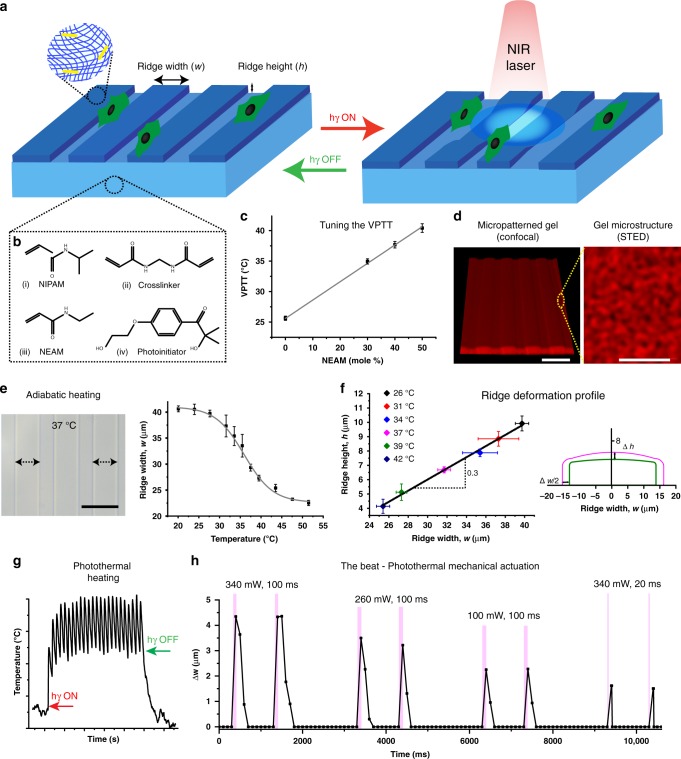
Thermoresponsive hydrogels that respond to a light trigger for rapid actuation. **a** Schematic showing the concept of actuating gels. Soft and patterned thin-film hydrogels are prepared from a thermoresponsive polymer. The gel contains AuNRs and is covalently bound to a glass coverslip. When a NIR laser shines on the gel, the AuNRs convert light to heat and the temperature of the gel locally increases to collapse the gel; therefore, a pulsing laser results in actuating (beating) gels. **b** The gel films are prepared with 57 wt% NIPAM and NEAM monomers at different ratios, 1 mole % crosslinker (N, N’-methylenebisacrylamide), and 1 mole % photo-initiator (2-hydroxy-4-(2-hydroxyethoxy)-2-methylpropiophenone). **c** The  VPTT of the gels is tuned to 37 °C with a NIPAM/NEAM ratio of 60/40 mole %, *n* = 3. **d** 3D confocal stack of a hydrogel film with 25 µm ridges and 25 µm valleys, scale bar = 50 µm. STED microscopy of gels at 37 °C, showing the microstructure of the gel, scale bar = 1 µm. **e** A representative brightfield image of a 60/40 % NIPAM/NEAM patterned gel at 37 °C. The VPTT for gels is calculated from changes in the ridge width at different temperatures in thermal equilibrium and adiabatic conditions, scale bar = 50 µm, *n* = 3. **f** The actuation beat is represented by the gel deformation with respect to the ridge width and height at different temperatures under adiabatic conditions, showing Δh/Δw ~ 0.3, *n* = 3. The illustration (drawn to scale) depicts the ridge shape at 37 and 39 °C. **g** Schematic of photothermal heating resulting in temperature changes of the gel that are synchronised with the laser pulses. **h** Due to local temperature changes, small changes in the ridge width (mechanical deformation, the beat) are also synchronised with the laser. The amplitude is defined as the maximum change in ridge width and is controlled by reducing laser power from 340 to 260 to 100 mW for a 100 ms pulse at 1 Hz or by changing the pulse duration to 20 ms at 340 mW at 1 Hz. Error bars represent standard deviation

Here, we demonstrate a light-responsive hydrogel platform, which supports unrestricted cell growth and reversibly applies precise and user-defined mechanical forces on selected cells under physiological conditions. This approach comprises a soft and patterned thin-film ECM-mimetic hydrogel prepared with a ratio of 60/40 mole % N-isopropyl acrylamide (NIPAM) and N-ethyl acrylamide (NEAM) to achieve a volume phase transition temperature (VPTT) around 37 °C, AuNRs for photothermal heating, and a fibronectin/collagen I surface coating for cell attachment (Fig. 1a). Cells grow at ~36 °C and the gel exhibits local mechanical actuation (beats) with a specific amplitude for an indefinite length of time at variable frequencies up to 10 Hz using a near infra-red (NIR) laser as light trigger. Precise, transient forces generated can be controlled at a user-defined sub-cellular or sub-population scale that is not pre-determined during gel fabrication. The direction of applied forces can be changed by the pre-defined topography of the hydrogel. As proof of principle, a subset of L929 fibroblast cells is actuated, resulting in changes in cell migration and nuclear translocation of the mechanosensor protein myocardin related transcription factor A (MRTFA), depending on the duration, amplitude and frequency of actuation. The Yes-associated protein (YAP) remains in the nucleus before, during and after actuation.

## Results

### Defining the photothermal actuation beat

Thin hydrogel films with micropatterns that are confined to a glass coverslip are prepared with a NIPAM/NEAM molar ratio of 60/40% to achieve a VPTT ~37 °C in the presence of cell culture media (Fig. 1b, c, Supplementary Fig. 1, 2, Supplementary Note 1). Unless otherwise mentioned, these gels are used for all experiments and referred to as gels or hydrogels. The gels are formed on a silicon wafer with continuous arrays of elevated ridges of 25 µm width and 2.7 µm height, separated by 25 µm valleys, while their internal structure is porous (Fig. 1d).

Adiabatic heating (in thermal equilibrium) of the entire gel (Fig. 1e, f) results in deformation of the microtopography. The ridge width (w) shrinks from ~40 to 24 μm over a temperature range of 26–42 °C, while the ridge height (h) shrinks from ~10 to 4 μm (Fig. 1f). Ridge deformation is predominant in the lateral direction (Δw) as compared to the axial direction (Δh) ((Δh)/(Δw) ~0.3), in contrast to flat confined gels^30^ (Fig. 1f, Supplementary Note 2). A comparison of the lateral swelling ratio for the ridges (~1.8) with that for free-standing hydrogel discs (~1.6), (Supplementary Fig. 3, Supplementary Table 1) suggests that the confinement of the patterned hydrogel film, tethered to a glass coverslip does not significantly affect the swelling and shrinking ability of the ridges. In contrast to reported literature on confined gels^30^, creases are avoided by keeping a ridge filling fraction of 0.5 (Supplementary Fig. 4, Supplementary Note 2). To render the gels photoresponsive, PEG-stabilised AuNRs are added to the precursor solution before gelation at a concentration of ~3.6 AuNRs/µm^3^, corresponding to a volume fraction of 0.004% AuNRs (Supplementary Fig. 5). Due to the photothermal effect of the AuNRs, the temperature of the irradiated gel area increases, resulting in a local gel collapse. In the case of stroboscopic illumination using a NIR laser, rapid gel actuation is achieved in the milli-second range. While adiabatic heating reflects the maximum extent of mechanical deformation and does not show spatial selectivity, local photothermal heating results in local temperature shifts that cause volume transitions (non-equilibrium), corresponding to the stroboscopic input. (Fig. 1g, h). The laser pulses (frequency and duty cycle) and power are varied to alter the amplitude and frequency of actuation. Spatially controlled actuation of the gel is achieved in a user-defined region of the gel (Movie 1, 340 mW, 1 Hz, ON time 100 ms, higher magnification in Movie 2). The non-irradiated region of the gel does not actuate and thus functions as a control region. The ridge lines are considered as reference points to measure the total change in ridge width, with the maximum change during one laser pulse defined as the amplitude, which changes from ~4.3 to 1.6 µm for different laser settings (100 ms and 20 ms laser ON time, Movie 2 and 3, respectively, Fig. 1h, Supplementary Table 2). During the recovery time when the laser is off, the gel cools down and re-swells before the light is back on (Supplementary Note 3). As the mesh size of similarly crosslinked hydrogels is reported to be ~5 nm, it can safely be assumed that the AuNRs are trapped inside the gel^31^. Inductively coupled plasma-optical emission spectroscopy (ICP-OES) confirms that the AuNRs do not leach out from the crosslinked hydrogel after swelling for 3 days (Supplementary Fig. 5e). The presence of these low amounts of AuNRs inside the gel does not affect the volume phase transition behaviour (Supplementary Fig. 6).

**Fig. 2:**
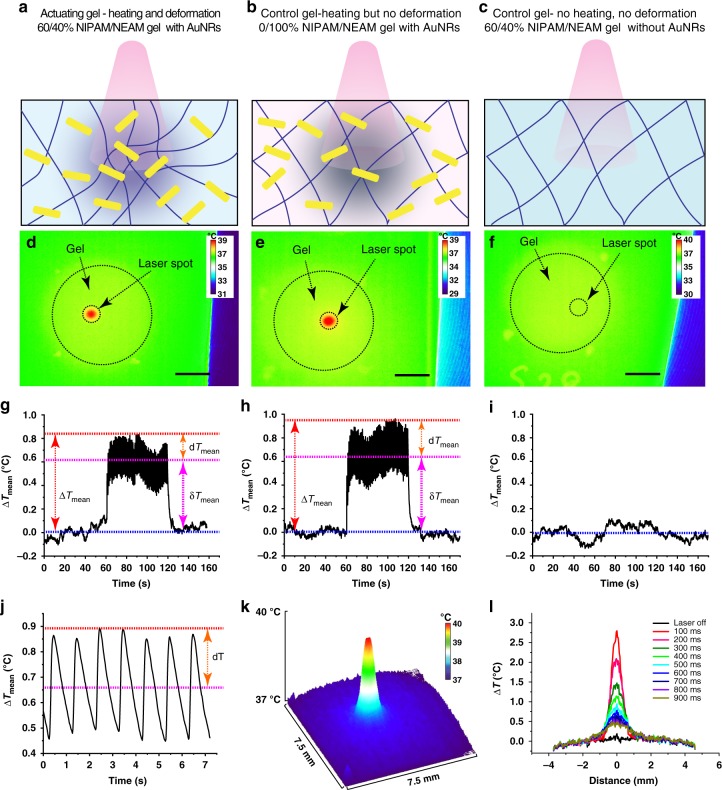
Temperature modulation during actuation does not exceed hyperthermia temperature. A schematic of **a** 60/40 NIPAM/NEAM gels with AuNRs, which actuate and show temperature changes when irradiated with NIR light, **b** 0/100 NIPAM/NEAM gels with AuNRs, which show a temperature gradient but do not actuate when irradiated with NIR laser, **c** 60/40 NIPAM/NEAM gels without AuNRs, which do not actuate or generate a temperature gradient when irradiated with NIR laser. The temperature gradient is depicted with color shading and the deswelling is shown by changes in the mesh size (not to scale). **d**–**f** Representative thermal images obtained during photothermal heating of the gels for **a**–**c**, respectively, scale bar = 3 mm. The laser is turned on at ~60 sec (340 mW, 1 Hz, 100 ms laser ON time) and turned off at ~120 sec. **g**–**i** Temperature profiles for the pulsed laser, calculated from images as depicted in **d**–**f**, respectively. **j** The mean temperature of the irradiated portion of the gel oscillates with the laser frequency. **k** A surface plot of the gel temperature, showing the spatial control of actuation. **l** The spatial temperature profile changes with the laser pulse, which is turned on at t = 0 ms. (340 mW, 1 Hz, 100 ms ON time)

To decouple the effect of mechanical actuation from photothermal heating of the gel, two controls are performed; one to investigate the effect of light exposure and one to test the impact of photothermal heating, both without mechanical actuation at ~36–37 °C. For the first control, 60/40 NIPAM/NEAM gels without AuNRs are used (light, no heat, no deformation), while the second control comprises gels made with 100% NEAM (LCST ~82 °C) and AuNRs (3.6 AuNRs/μm^3^) (light + heat, no deformation). The local temperature changes that occur when the gels are irradiated with a NIR laser at 340 mW, 1 Hz and 100 ms laser ON time are measured with an IR camera (Fig. 2a–c, Supplementary Fig. 7a). The gels with AuNRs show photothermal heating, while no such effect is observed for the control gel without AuNRs (Fig. 2d–f). The temperature corresponding to the thermograms is quantified over time (Fig. 2g–i), demonstrating a mean total increase (averaged over the irradiated area and compared to the averaged ground state before light exposure) ΔT_mean_ of ~0.9 °C. The photothermal effect leads to a new thermal equilibrium state during actuation (δT_mean_ ~0.6 °C higher than the ground state before light exposure) around which the gel temperature oscillates (dT_mean_ ~± 0.3 °C) during pulsing, synchronised with the laser pulse (Fig. 2g–j, Movie 4, 5). The ridge deformation amplitudes remain constant for 22 h of actuation, which suggests that the temperature does not increase when pulsed for a prolonged time and that it is possible to stay close to the physiological temperature by minimising dT_mean_ during actuation. The maximum temperature increase, ∆T_max_, (determined from the maximum temperature measured in the irradiated portion of the gel compared to the temperature before light exposure) is ~3.0 °C, while the maximum temperature oscillations around the new equilibrium (dT_max_) are ~± 1.5 °C, keeping the maximum temperature (due to positive fluctuations) below 39.0 °C and thus below the temperature of heat shock for cells^32^ (Supplementary Fig. 7b,c, Supplementary Note 4). This maximum temperature is not experienced throughout the actuated region but spatially confined and in tandem with the laser pulses. The temperature returns to the new equilibrium in ~700 ms after a 100 ms pulse is fired (Fig. 2l, Movie 5), which is in agreement with the estimated temperature based on a thermal calibration curve (Fig. 1e, Supplementary Note 4). The experimental temperature observations are supported by simulation data using finite element analysis (Supplementary Fig. 8, Supplementary Note 5, Supplementary Table 3), where a total mean temperature increase (dT) of ~1.8 °C is expected due to NIR irradiation. To test the effect of temperature on cell proliferation, cells are grown on tissue culture polystyrene (TCPS) for 24 h, revealing no significant difference between 30 and 40 °C, while cell proliferation at 25 and 45 °C is reduced (Supplementary Fig. 9a). Exposure to 44–45 °C for 2 h is known to cause thermal shock leading to over expression of the heat shock protein (Hsp 70)^33^. Stress granules made of non-translating mRNA and P bodies that contain proteins, which decay mRNA, are generated in response to oxidative, osmotic, heat or UV irradiation stresses or viruses^34^. Therefore, the levels of Hsp 70, along with stress granules and P bodies, are quantified and compared for actuated vs. non-actuated cells by immunofluorescence, demonstrating no significant difference between both conditions (Supplementary Fig. 9), suggesting that cells are not unduly mechanically or thermally stressed due to actuation.

### Cell migration and mechanotransduction affected by actuation

Cells are seeded on fibronectin coated 60/40 NIPAM/NEAM hydrogel films and allowed to adhere for ≥12 h, after which the gels are actuated (Supplementary Fig. 10). Fibronectin is covalently linked to the gel using optimised conditions (Supplementary Fig. 11, Supplementary Note 6). An exposure to NIR light (340 mW, 1 Hz, 100 ms ON time) during the time course of 22 h does not affect cell viability as shown using a live-dead assay for 60/40 NIPAM/NEAM gels in the actuated and non-actuated area (Supplementary Fig. 11e). A representative brightfield 12 h time-lapse video of cells on the surface of actuating hydrogels (340 mW power, 1 Hz, 100 ms ON time) is shown in Movie 6. Cell motility parameters are measured for individual cells on actuating gels and compared against cells on control, mechanically invariant gels of 100% NEAM, which do not actuate.

**Fig. 3:**
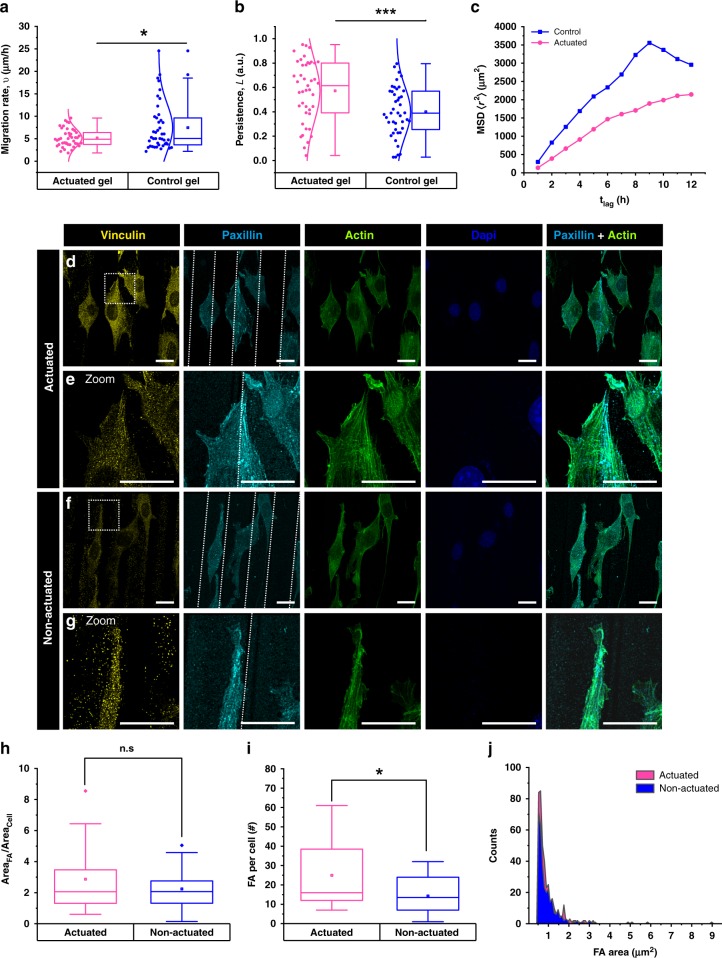
Linking actuation effects on cell migration to focal adhesions. **a**, **b** Box plots showing cell motility parameters on 60/40 NIPAM/NEAM gels as a result of gel actuation, in comparison to non-actuating 0/100 NIPAM/NEAM control gels, **a** the migration rate **b** the persistence. The end-to-end distance, the contour length and the projected contour length can be found in Supplementary Fig. 12. **c** The Mean Squared Displacement as a function of lag times. (*n* = 2, *N* ≥ 43 cells). **d**–**g** Effect of actuation on the formation of focal adhesions is investigated using vinculin and paxillin immunofluorescence. **d**, **e** Cells are actuated for 12 h (340 mW, 1 Hz, 100 ms laser ON time), **e** shows a magnification of the dotted square in **d**. The edges of the ridge are marked by white dotted lines, showing an increase in focal adhesions along the ridge edges. **f**, **g** Control cells that are not irradiated with the laser and hence, not subject to actuation, with **g** showing a magnified image of the dotted square marked in **f**. **h** The total area occupied by all focal adhesions per cell. **i** The total number of focal adhesions per cell for the actuated and the non-actuated conditions are represented by box plots, which increase due to actuation. **j** Histogram of the area of focal adhesions for the actuated vs. the non-actuated cells (*n* = 3, *N* ≥ 17 cells). In the box plots, the interquartile range (IQR) between the first and the third quartiles is indicated by the box, while whiskers denote 1.5 IQR. The hollow square, the horizontal line, and the filled dots represent the average, the median, and the outliers, respectively. On the left of the box plot, all data points are shown, the normal distribution curve serves to guide the eye. *, **, *** are determined using one way ANOVA or Welch test, depending on the homogeneity of variances, and represent statistical significance at *p* < 0.05, 0.01 and 0.001, respectively. **d**–**g** Scale bar = 20 µm

On both the actuated and control gels, cells move erratically along the ridges (Fig. 3a–c, Supplementary Fig. 12, Supplementary Note 7) with a reduced migration rate (~5 and ~7.5 µm/h for the actuated and the control gel, respectively) and increased persistence (~0.6 in the case of actuated cells while ~0.4 for the control), while the end-to-end distance of the trajectories (the net displacement of cells from the initial position to the final position) is not affected by actuation (mean ~50 μm) in ~12 h, (Supplementary Fig. 12). Persistence is a good indicator of the effect of structural guidance provided by the gel topography; actuating gel topography further enhances cell movement along the topography. The projected contour length, (the distance travelled by cells in the direction of the ridges) remains higher for non-actuated gels (~75 μm as compared to ~50 μm with actuation, ~33% decrease) due to the overall higher migration rate. To mechanistically explain slower cell migration on actuating surfaces, the focal adhesions (FA) on cells actuated for 12 h (340 mW, 1 Hz, 100 ms laser ON time) are stained against vinculin and paxillin and compared to non-actuated cells (Fig. 3d–j). Actuation leads to an increase in the number of focal adhesions per cell (from ~15/cell to ~24/ cell, ~60% increase upon actuation), while the size of focal adhesions and the net area (Area_FA_/Area_cell_) occupied by focal adhesions per cell remain the same in both the cases. This suggests that actuation leads to greater cellular adhesion to the substrate through more focal adhesions resulting in a larger cellular spread area.

During cell adhesion and migration, actin filaments in the cytoskeleton undergo dynamic changes. Globular actin (G-actin) is polymerised to form actin filaments (F-actin) and generates the forces necessary to establish focal adhesions and protrude lamellipodial or filopodial extensions^35^. The myocardin related transcription factor A (MRTFA) is known to be associated with cytoskeletal dynamics and is a direct measure of the G-actin concentration in the cell, as it releases G-actin and shuttles to the nucleus^35–37^. Another protein that has occupied a centre-stage in understanding mechanobiology is the Yes-associated protein (YAP), which can respond to a variety of mechanical cues, such as substrate rigidity and topologies affecting cell geometry^38^. The intracellular localisation of YAP is considered to depict the substrate stiffness, as it is mostly localised in the nucleus when cells are grown on stiff elastic substrates (>30 kPa) and in the cytoplasm when cells are grown on soft elastic substrates (≤5 kPa)^39^.

After actuation, cells are immunostained against MRTFA and YAP. In the case of MRTFA, a distinct translocation from the cytoplasm to the nucleus is observed, depending on the laser settings and duration of exposure. The cells growing outside the exposed region on the same gel function as control cells and do not show MRTFA shuttling (quantified to give Nuclear MRTFA%, Supplementary Fig. 13). On the control gels, 0/100 NIPAM/NEAM with AuNRs (heating, no mechanical deformation) and 60/40 NIPAM/NEAM gels without AuNRs (no heating, no mechanical deformation), MRTFA also remains in the cytoplasm (Supplementary Fig. 14). Additionally, to show that MRTFA is not affected by temperature, cells are cultivated at 36 °C and 39 °C under static conditions on top of the hydrogel film (Supplementary Fig. 15). In both cases, MRTFA is present in the cytoplasm. These control conditions assist in decoupling the effect of mechanical actuation from other factors, such as gel topography, light, temperature and photothermal heating, with only minimal changes in stiffness upon actuation.

**Fig. 4:**
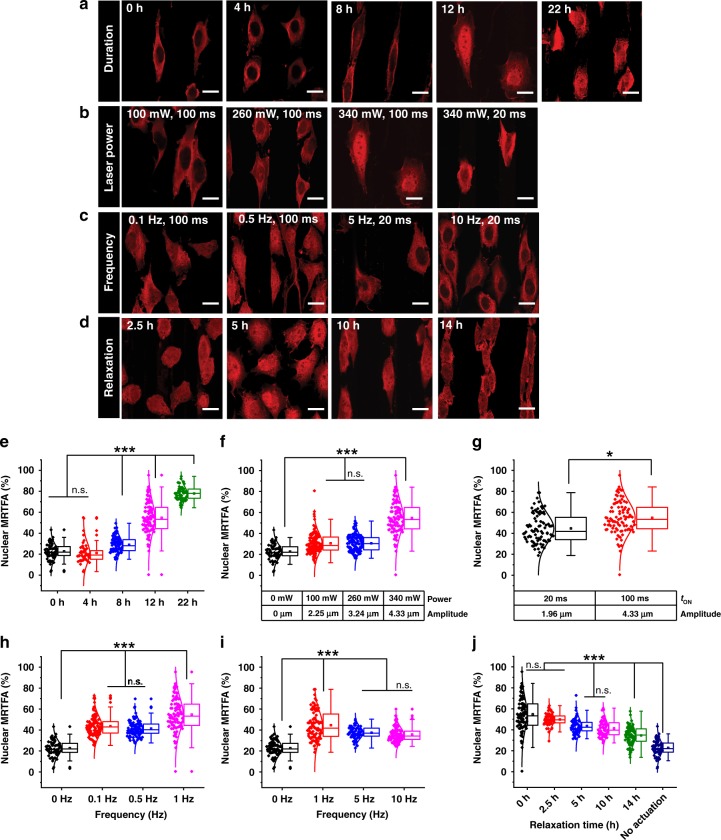
Immunofluorescence images showing the distribution of MRTFA in the cells. **a** Cells are actuated for 0, 4, 8, 12 and 22 h (340 mW, 1 Hz, 100 ms laser ON time), **b** The actuation amplitude is varied by using a laser power of 100 mW, 260 mW or 340 mW (1 Hz, 100 ms ON time, 12 h actuation), or by reducing the laser pulse to 20 ms at 340 mW laser power and 1 Hz frequency. **c** Cells are actuated at 0.1 Hz and 0.5 Hz at 340 mW (100 ms ON time,12 h actuation), while higher frequencies of 5 Hz and 10 Hz are obtained using a shorter ON time of 20 ms, keeping the laser power constant (340 mW). **d** After actuating for 12 h (340 mW, 1 Hz, 100 ms laser ON time), actuation is ceased and cells are allowed to relax for 2.5, 5, 10 and 14 h. **e**–**j** The nuclear MRTFA % is measured to assess the effect of **e** duration of actuation, **f** actuation amplitude by changing laser output, **g** actuation amplitude by changing the ON time of the laser pulse, **h** frequency when ON time = 100 ms, **i** frequency when ON time = 20 ms and **j** relaxation. Scale bar = 20 µm. The box plots show the nuclear MRTFA % for each cell (*n* ≥ 3, *N* ≥ 60 cells, except **e**, where *N* ≥ 48 cells). In the box plots, the interquartile range (IQR) between the first and the third quartiles is indicated by the box, while whiskers denote 1.5 IQR. The hollow square, the horizontal line, and the filled dots represent the average, the median, and the outliers, respectively. On the left of the box plot, all data points are shown, the normal distribution curve serves to guide the eye. *, **, *** are determined using one way ANOVA or Welch test, depending on the homogeneity of variances, and represent statistical significance at *p* < 0.05, 0.01 and 0.001, respectively

When the duration of actuation is varied from 4, 8, 12 to 22 h, the nuclear signal of MRTFA gradually increases when the ridges shrink and swell with an amplitude of ~4.3 µm (~14% deformation, 340 mW, 1 Hz, 100 ms ON time). Fluorescence intensity quantification of MRTFA in the nucleus and cytoplasm (Fig. 4, Supplementary Fig. 16) shows that after 4 h of actuation, nuclear MRTFA accumulation is not yet visible but nuclear translocation starts around 8 h. The MRTFA levels in the nucleus further increase after 12 and 22 h. For non-actuated cells, the distribution of MRTFA is uniform across the cell population, while a broader distribution is visible upon actuation. Although accumulation in the nucleus is higher at 22 h (75%), the 12 h time point is further used throughout this study as the doubling time of the L929 cell line is ~24 h.

To investigate the effect of amplitude on MRTFA translocation, the laser output power is decreased from 340 to 260 to 100 mW, while maintaining the frequency of actuation at 1 Hz (100 ms ON time) for a duration of 12 h. A laser power of 100 or 260 mW, corresponding to an actuation amplitude of 2.2 or 3.2 µm, respectively, causes less MRTFA shuttling to the nucleus with nuclear MRTFA levels of ~30% in both cases compared to ~50% in the case of 340 mW, which is still significantly higher than for non-actuating controls (~20%) (Fig. 4b, f, Supplementary Fig. 17). Another way to control the amplitude of oscillation is by varying the duration of the pulse (ON time), for example a reduction from 100 to 20 ms (340 mW, 1 Hz) leads to an amplitude of ~2.0 µm instead of ~4.3 µm and less nuclear accumulation (~40% instead of ~50%) (Fig. 4b, g, Supplementary Fig. 18). Interestingly, a similar reduction in amplitude by lowering the laser power to 100 mW (1 Hz, 100 ms ON time) has less effect on MRTFA translocation.

For an ON time of 100 ms, and frequencies of 1, 0.5 and 0.1 Hz, respectively (laser output 340 mW, 12 h) (Fig. 4c, h, Supplementary Fig. 19), a frequency of 1 Hz results in the highest nuclear accumulation of ~50%, compared to ~40% for 0.5 or 0.1 Hz. The ON time is reduced to 20 ms to achieve frequencies of 1, 5 and 10 Hz (Fig. 4c, i) where 1 Hz leads again to the highest nuclear MRTFA levels (~45%, a bit less than for 100 ms ON time). Therefore, in these experiments, the most prominent effect is always seen at a frequency of 1 Hz.

**Fig. 5:**
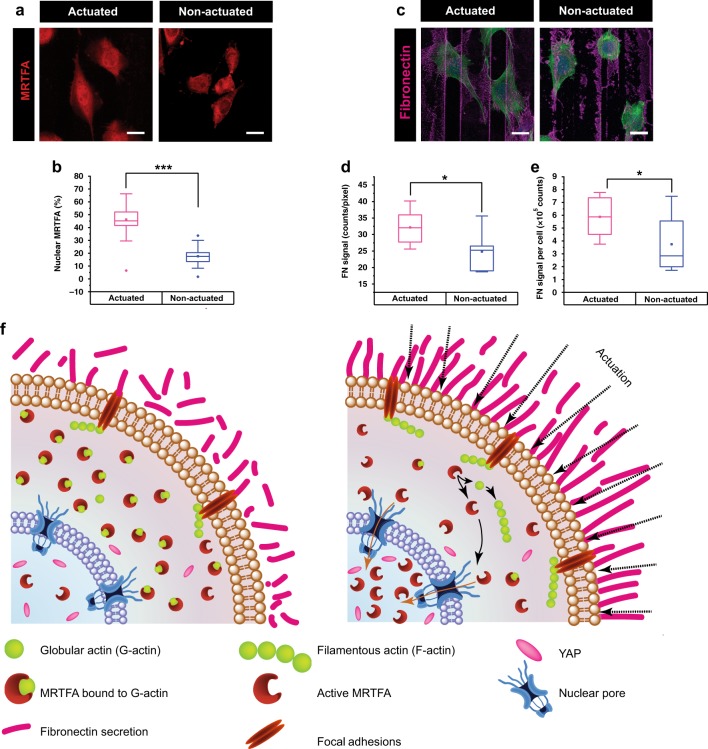
The effect of actuation on mechanosensitive proteins MRTFA and YAP, and the suggested mechanism. **a** Immunofluorescent images of MRTFA stained cells grown on collagen I coated gels (60/40 % NIPAM/NEAM gel with AuNRs) that are present in the actuating area (340 mW power, 1 Hz, 100 ms laser ON time, top row) showing nuclear localisation and in the non-actuating area where MRTFA is in the cytoplasm, **b** Quantification of the nuclear percentage of MRTFA (*n* = 3, *N* ≥ 60 cells). **c** Immunofluorescent images of cells grown on collagen I coated gels shows more aligned fibronectin secretion when cells are actuated, *n* = 3, *N* ≥ 211 cells) (green = actin, blue = nucleus, scale bar = 20 µm). **d**, **e** Quantification of the fibronectin signal shows more fibronectin in the actuated region as well as more fibronectin production per cell by actuated cells. **f** A schematic depicting the suggested mechanism. When the hydrogel film actuates, cells experience forces that are transduced to the nucleus via F-actin filaments, formed by G-actin polymerisation. When G-actin is released to make F-actin, MRTFA translocates from the cytoplasm to the nucleus. Upon cessation of actuation, MRTFA returns back to the cytoplasm. YAP, on the other hand, does not show shuttling behaviour in response to actuation of this hydrogel film but is already present in the nucleus due to the stiffness of this gel (~30 kPa under cell culture conditions). In the box plots, the interquartile range (IQR) between the first and the third quartiles is indicated by the box, while whiskers denote 1.5 IQR. The hollow square, the horizontal line, and the filled dots represent the average, the median, and the outliers, respectively. *, **, *** are determined using one way ANOVA or Welch test, depending on the homogeneity of variances, and represent statistical significance at *p* < 0.05, 0.01 and 0.001, respectively

To check the reversibility of this translocation, irradiation (340 mW power, 1 Hz, 100 ms ON time) is stopped after 12 h and the gel is allowed to relax for different durations (Fig. 4d, j, Supplementary Fig. 20). After cessation of actuation for 2.5, 5, 10 and 14 h, the nuclear % of MRTFA slowly drops from 50 to 45 to 40 to 35%, respectively and approaches the initial value of 20%. Therefore, it appears that the return kinetics for MRTFA from the nucleus to the cytoplasm is slower than nuclear translocation. To rule out the possibility that the fibrillar nature of the fibronectin coating affects the cell response to actuation^40^, cells are actuated on collagen I coated gels, revealing a similar nuclear translocation of MRTFA (Fig. 5a, b, Supplementary Fig. 21). Interestingly, actuated fibroblasts secrete more fibronectin (~33 counts/px), which is also aligned, as compared to the random nature and lower concentration of fibronectin secreted by non-actuated cells (25 counts/px) (Fig. 5c–e, Supplementary Fig. 22). These results are in agreement with the MRTFA nuclear shuttling, which is known to activate focal adhesion stabilising genes and reduce cell migration^41^. They suggest that the increase in fibronectin production in the case of actuating cells may contribute to long-term environmental changes that affect the cells further, for example the increase in MRTFA accumulation in the nucleus at later time points (~8 h) and the fact that, after turning off the light, the MRTFA only partially shuttles back to the cytoplasm.

On the other hand, when immunostaining for YAP, it is clear that YAP is located in the nucleus for both the actuated and non-actuated gel (340 mW power, 1 Hz, 100 ms ON time, 12 h, Supplementary Fig. 23). This may be explained by the stiffness of the hydrogel film at 37 °C near the VPTT (E ~30 kPa, AFM, Supplementary Fig. 24, Supplementary Note 1), promoting nuclear accumulation of YAP, which is also similar to the rigidity threshold for unfolding of talin^39,42^. This argument is supported by our control experiments, where YAP is located in the nucleus when cells are grown on stiff TCPS (E = 2 GPa)^43^ and in the cytoplasm when cells are grown on ultra-soft 1 wt % poly (ethylene glycol) (PEG) gels (G’ = 20 Pa)^44^, while under these circumstances, MRTFA is always found in the cytoplasm (Supplementary Fig. 25)

## Discussion

Soft tissues are often subjected to shear stress and cyclic mechanical strains^12^. While these forces are essential in maintaining a healthy physiology of different organs and tissues, a deviation leading to abnormal mechanical forces can result in disease. Therefore, in vitro platforms are required to replicate mechanical forces on cells and tissues to understand the key mechanosensor molecules involved in mechanotransduction and signalling pathways. This work demonstrates a smart hydrogel platform that can actuate to mechanically stimulate cells in a cyclic, user-defined manner. Since the modulus of the gels is sufficiently low, frequencies as high as 10 Hz are achieved, which is up to 2 orders of magnitude higher than other stretching systems reported in literature (0.1 Hz)^13^. The local actuation of the gel depends on the heat generation by the AuNRs, which is governed by the optical properties and concentration of the AuNRs and the intensity of the laser spot on the sample (laser fluence)^45^. The AuNR concentration of 3.6 AuNRs/µm^3^ does not affect the VPTT but is sufficiently high to enable the use of relatively low laser flux and laser on time (0.7–3.6 × 10^3^ W/cm^3^ for 20–100 ms), which is 2 orders of magnitude less than the 3.6 × 10^5^ W/cm^3^ for up to 5 min reported in literature^13^. Based on measurements, ~2.5–7% relative displacements are achieved with a δT_mean_ of ~0.1–0.6 °C, respectively (Supplementary Fig. 26, Supplementary Table 2, Supplementary Method 8).

Due to actuation of the hydrogel film, the cell migration rate is reduced, while the persistence of cells increases. In addition, MRTFA shuttles to the nucleus after 8 h depending on the frequency (maximum effect at 1 Hz), amplitude, and duration of actuation. It is likely that 1 Hz is a threshold value for the frequency, at which saturation of the cellular response occurs. Below this frequency, there may be limiting factors, such as insufficient reorganisation of the actin cytoskeleton and focal adhesions at work, while above this threshold, the internal machinery may be unable to react to the fast time scale^10^. According to the molecular clutch model, focal adhesions are given the analogy of a molecular clutch. A clutch is engaged when the actin network is coupled to the ECM through integrin binding, resulting in the formation of focal adhesions^46^. It has to break to enable cell migration. In the light of the mechanosensitive molecular clutch model^46,47^, more FAs observed on actuated cells correspond to more engaged clutches, which suggests talin unfolding succeeded by vinculin unfolding. As the gel modulus here is comparable to the talin unfolding threshold (~30 kPa, regarded as ‘stiff’ by the cell^42,46^), the presence of more focal adhesions upon actuation, suggests a further enhancement of talin unfolding due to actuation. It is well known that on stiff substrates, engaged clutches reach their breaking strength easily to facilitate cell migration. As less cell migration is observed on the actuating gels, it can be postulated that the rate of load transmission to the clutch is slowed down and the breaking strength is not reached as easily, although this hypothesis warrants further investigation in follow-up studies. Interestingly, more FAs are observed along the ridge edges, which are the sites of actuation.

This suggests the need for additional polymerisation of actin in response to the mechanical stresses experienced by the cells and corroborates with previous reports that MRTFA plays an important role between mechanical stresses and mechanotransduction to the nucleus. YAP, on the other hand, is unaffected by actuation and is always in the nucleus when the cell is sufficiently spread. The ‘nuclear pore’ theory provides a nexus between nuclear accumulation of proteins and substrate stiffness^39,48^. Nuclear entry of protein molecules is promoted by nuclear flattening that leads to an enlargement of nuclear pores and depends on the 3D size of the protein. As nuclear flattening occurs when cells spread, this is greatly affected by substrate stiffness. At 36 °C, the present hydrogel film has a modulus of ~30 kPa, leading to nuclear pores that are sufficiently large for YAP (65 kDa), which is already mainly located in the nucleus on static gels. On the other hand, the larger protein MRTFA (160 kDa) only enters the nucleus upon actuation (Fig. 5). This suggests two possibilities: the nuclear pores are large enough before actuation to facilitate MRTFA entry or they undergo further enlargement during actuation. As no difference in cell spreading is observed between the actuated and the non-actuated gels, the first possibility is more likely.

Cells ‘feel’ the repetitive small deformations (~1.6–4 µm), which manifest in changes in focal adhesions and their migration rate, driven by intracellular molecular pathways. User-defined forces are locally exerted on gels and affect cell behaviour in a mechanobiological manner when applied for multiple hours, while gel topography, stiffness and surface coating can be independently varied. This allows for the identification of (disrupted) mechanosensor circuits, as mechanical forces are decoupled from the physical and biochemical environment of cells. To the best of our knowledge, this is the first system that can exert such stresses on a spatially selected cell population for a prolonged period of time.

## Methods

### Materials

N-isopropyl acrylamide (NIPAM, 97%), N-ethyl acrylamide (NEAM, 99%), photo-initiator (2-hydroxy-4-(2-hydroxyethoxy)-2-methylpropiophenone, 98%), crosslinker (N, N’-methylenebisacrylamide, 99%), acetone, isopropanol, 3-(trimethoxysilyl) propyl acrylate (92%) and dimethyl sulfoxide (DMSO) are purchased from Sigma–Aldrich. Fluorescent hydrogels are prepared by using fluorescein-o-acrylate (Sigma–Aldrich) or methacryloxyethyl thiocarbomyl rhodamine B (Polysciences) and moulds for compression test are made from poly(dimethylsiloxane) (PDMS, Sylgard 184, Dow Corning). All solvents are purchased from Sigma–Aldrich unless otherwise mentioned. Deionised water (0.1 μS/cm, ELGA Purelab-Plus), sodium borohydride (NaBH_4_, 99%), cetyltrimethylammonium bromide (CTAB, 99%) hydrogen tetrachloroauric (III) acid (HAuCl_4_,), ascorbic acid (99%) silver nitrate (AgNO_3_, 99.99%), chloroform are used for the production of gold nanorods. Thiol functionalised polyethylene glycol (PEG) polymer (HS-PEG-OH, Mw = 3000 Da, Iris Biotech) is used for the modification of the AuNRs. L929 mouse-derived fibroblasts are cultured in RPMI medium (Gibco), supplemented with 10% fetal bovine serum (FBS, Biowest) and 1% antibiotics/antimycotics (Gibco). Fibronectin from human plasma (Sigma–Aldrich, F2006) or Collagen 1 (Thermo Fisher A1064401) is dissolved in endotoxin-free water and used for coating the gels using sulfosuccinimidyl 6-(4’-azido-2’-nitrophenylamino)hexanoate (Sulfo-SANPAH) (Thermo Fischer 22589) as a covalent linker. The MTS (3-(4,5-dimethylthiazol-2-yl)-5-(3-carboxymethoxyphenyl)-2-(4-sulfophenyl)-2H-tetrazolium) assay (Promega G5421) is used to quantify the number of viable cells. Anti-mouse MRTFA primary antibody (Santacruz biotech, sc398675) is used at a dilution of (1:100), while anti-mouse Alexa Fluor 633 from Thermo Fischer (A 21050) is used as the secondary antibody, dilution 1:100. YAP primary antibody (Cell Signaling Technologies, 4912 S) is used at a dilution of 1:100 with anti-rabbit secondary AF 488 (Thermo Fisher, dilution 1:100). Cell nuclei are stained with DAPI (4′,6-diamidino-2-phenylindole, while the actin cytoskeleton is stained using cytopainter iFluor-594 (abcam ab 176757, dilution 1:400). Anti-fibronectin from rabbit (Sigma–Aldrich F3648), anti-paxillin from rabbit (Sigma–Aldrich SAB4502553) and anti-vinculin from mouse (Sigma–Aldrich V9264) is employed at a dilution of 1:100.

### Preparation of hydrogels

The hydrogels are fabricated using a silicon mould template replication polymerisation technique. Since free-standing thin gels do not retain their shape, gel films are tethered to a glass coverslip silanised with (3-(trimethoxysilyl) propyl acrylate silane) (Supplementary Method 1). Hydrogels are fabricated by co-polymerisation of N-isopropyl acrylamide (NIPAM) and N-ethyl acrylamide (NEAM). NIPAM is recrystallised from hexane and NEAM is passed through a neutral alumina column before use. A polymer precursor solution, which contains the monomers NIPAM and NEAM, crosslinker and initiator in DMSO forms a thin film between an acryl silanised coverslip and a microstructured silicon wafer with continuous grooves and ridges, both 25 µm wide and 2.7 µm deep (Supplementary Fig. 1, 2, Supplementary Note 1). To obtain a VPTT near the physiological temperature of 37 °C, hydrogels with different ratios of NIPAM (LCST ~32 °C) and NEAM (LCST 82 °C)^49^ are prepared. The molar ratios of NIPAM and NEAM are varied from 100/0, 70/30, 60/40, 50/50 and 0/100 mole % NIPAM/NEAM to vary the volume phase transition temperature (VPTT) of the gel. For a 60/40 molar ratio of NIPAM/NEAM, 0.2 g NIPAM and 0.12 g NEAM is added to 333 μL DMSO along with 6.6 mg photo-initiator (Irgacure 2959) and 4.5 mg crosslinker (N,N’-methylenebisacrylamide, BIS), resulting in a VPTT around the physiological temperature (37 °C). Upon UV polymerisation, a thin gel film is covalently attached to the modified glass coverslip. The VPTTs are determined by measuring the dimensions of the ridge widths on the gels in the presence of cell culture medium and defined as the inflection point of the sigmoid curve. PEG-functionalised gold nanorods (AuNRs) in DMSO are added in such a way that an optical density (OD) of ~95 with a AuNR number density 3.6/μm^3^ is achieved at a wavelength of 808 nm for a path length of 1 cm. The amount of DMSO is compensated to account for the AuNRs added and achieve a gel precursor solution at ~57 w/v % monomer.

Fabrication of patterned or flat hydrogels is performed in the glovebox in a low oxygen environment (<0.40%). Drops of gel precursor solution (1.5 μL each) are placed on top of the silicon wafer and an acryl silanised coverslip is placed on top of the drop such that the precursor solution is present in the interspace between the silicon wafer and the coverslip.

The gels are cured for 30 min under UV light (Konrad Benda lamp 366 & 254 nm, 8 W). After curing, they are immersed overnight in excess de-ionised water at ~25 °C to remove DMSO. This facilitates hydrogel swelling and easy peeling of the gels from the patterned silicon surface. Fluorescent hydrogels are prepared by adding 0.4 mg fluorescein-o-acrylate or methacryloxyethyl thiocarbonyl rhodamine B (Polysciences Inc) in 100 μl of the gel precursor solution. The gels are washed thrice with excess DMSO to remove the unreacted monomers and then thrice with Milli Q water. The gels are sterilised in 70% ethanol for 30 min and washed with water thrice. Surface functionalisation of the gels with fibronectin is used for all photothermal analysis in this study, except for AFM measurements and ridge width analysis at thermal equilibrium for different temperatures to determine the VPTT.

### Synthesis and modification of AuNRs

AuNRs are synthesised by a seed-mediated growth method, as reported elsewhere^31,50,51^. A fresh seed solution is prepared by adding 0.60 ml of an aqueous ice-cold 0.010 M NaBH_4_ to a mixture of water (4.2 mL), 0.20 M CTAB (5.0 mL) and 0.0030 M HAuCl_4_ (0.83 mL) under vigorous stirring for 2 min. The growth solution is prepared by adding a mixture of 0.20 M CTAB (150 mL), 0.050 M ascorbic acid (3.1 mL) and 0.0080 M AgNO_3_ (3.3 mL) to 0.0010 M HAuCl_4_ (150 mL). 0.875 mL of seed solution is added to the growth solution at 25 °C in a water bath and stirred vigorously for 30 min. This is followed by the addition of 0.050 M ascorbic acid (2.0 mL) at a flow rate of 0.50 mL/h and stirring for another 30 min. The resultant brownish red solution containing AuNRs is centrifuged at 10,492 × *g* for 25 min in a centrifuge (Hereaus Multifuge X3R). The AuNRs that precipitate are resuspended in 2 mL water (0.1 μS/cm, ELGA Purelab-Plus), while the supernatant containing excess surfactant is discarded.

A 10 mL solution of the AuNRs, prepared as above, is re-dispersed in 100 mL water and mixed with 20 mL solution of 2.5 mM thiol functional PEG (HS-PEG-OH, Mw = 3000 Da, Iris Biotech) in ethanol under stirring. This solution is sonicated at 60 °C for 30 min and subsequently for another 3.5 h at 20 °C. The solution is stirred overnight and then extracted with chloroform thrice (~120 mL) to remove CTAB and free polymer. In a second purification step, the AuNRs are transferred to 25 mL DMSO and centrifuged at 9416 × *g* for 20 min. The supernatant is discarded and the solution containing concentrated AuNRs (2 mL) is diluted with DMSO and centrifuged three times. After the last centrifugation, the concentrated AuNR dispersion in DMSO is used for further experiments. The modified AuNRs have a longitudinal surface plasmon resonance at *λ* = 795 nm (UV-Vis spectrum, V-630, JASCO). They have an average length of 70 nm, width of 16 nm, and aspect ratio of ~4.4. The amount of AuNRs is quantified (Supplementary Method 2) and pre-mixed at a specific concentration (3.6 AuNRs/μm^3^) in the gel precursor solution before polymerisation.

### Determination of the volume phase transition temperature (VPTT)

The VPTT of non-surface functionalised gels is analysed by measuring the widths of the patterns in response to changes in temperature (Fig. 1). The patterned gels are placed inside a custom-made PDMS mold that is filled with cell culture medium and put on a Peltier stage, fixed to an optical microscope (Keyence, VHZ 100UR). A small temperature sensor (Pt, K100) is connected to a Keithley resistor and is used to measure and record the temperature of the medium at an interval of 1 s. Micrographs are captured after the gel is allowed to equilibrate for at least 15 min at different temperatures and analysed using ImageJ software. The ridge widths are calculated from the plot profiles.

### Laser actuation setup and exposure conditions

A custom-made setup, which can simultaneously irradiate the gels at the required wavelength, as well as observe the gel and cells, is designed to perform the experiments (Supplementary Fig. 10, Supplementary Method 3). The laser is mounted on an inverted microscope and focussed on the gel through a collimator. A port on the microscope (Zeiss Axiovert 100) is especially modified to connect a diode laser (λ_max_ 808 nm, maximum power 340 mW, Roithner Lasertechnik) through a fibre optic cable, while a collimator is used to focus the laser beam on the sample (footprint area ~1.2 mm^2^, major axis ~1.9 mm, minor axis 0.83 mm). An incubator chamber around the microscope ensures that standard cell culture conditions are maintained. The gels are mounted on a custom-designed sample holder, Supplementary Method 3). A low pass IR filter (Edmund Optics) is added in the optical path to prevent the IR light from reaching the high-speed camera (Hamamatsu ORCA-Flash 4.0 CMOS). The laser power reaching the gel is experimentally measured using a power meter (Thor Labs PM 400) and the intensity is calculated from the spot size observed on the gel. The laser footprint on the gel is kept the same for all experiments. Different laser settings are applied (Table 1) and an Arduino circuit is used as a variable trigger to control the laser. Brightfield images of the gels are acquired at a rate (10–100 fps) when the laser is pulsing. The actuation amplitude is determined as the maximum difference in ridge width during actuation using Image J software. The actuated region of the gel is identified using grid markings and all cells growing in the actuated zone are considered for analysis. Cells growing at least 200 µm away from the actuated regions are chosen randomly as control cells.

**Table 1:** Experimental parameters used for actuation

Parameter	Experimental conditions varied	Experimental conditions kept constant
Time of actuation (h)	0, 4, 8, 12, 22	@ 340 mW, 1 Hz, 100 ms laser ON time
Laser Pulse (ms)	0, 20, 100	@ 340 mW, 1 Hz, 12 h laser exposure
Laser output (mW)	0, 100, 260, 340	@ 1 Hz, 100 ms laser ON time, 12 h laser exposure
Frequency (Hz)	1, 0.5, 0.1	@ 340 mW, 100 ms laser ON time, 12 h laser exposure
Frequency (Hz)	1, 5, 10	@ 340 mW, 20 ms laser ON time, 12 h laser exposure
Relaxation (h)	0, 2.5, 5, 10, 14	After laser exposure @ 340 mW, 100 ms laser ON time, 12 h laser exposure

### Temperature measurements

An IR thermal imaging camera (FLIR A655 sc) is employed to measure the temperature changes due to photothermal heating (Supplementary Fig. 7a). For this purpose, the gel is sealed with cell culture medium in a secure seal spacer (Electron Microscopy Sciences, 70327) with glass coverslips (Marienfeld, 1.5 H) on both sides and is placed on top of a Peltier stage. The laser is incident obliquely from the top. Prior to measuring samples, the thermal camera is calibrated before the measurement using a pre-calibrated Peltier stage, whose temperature is set at 30 °C (lower limit) and 43 °C (upper limit), the emissivity value is set at 0.98. The IR camera is calibrated assuming a linear fit between the radiance values at the upper and lower limit temperatures between which the calibration is valid. The thermal images are recorded at a speed of 50 fps before (0–60 s), during (61–120 s) and after pulsing (121–160 s). Gels are pulsed with a frequency of 1 Hz, ON time of 100 ms, and 340 mW laser power. The temperature changes for control gels (0/100 NIPAM/NEAM gel with AuNRs and 60/40 NIPAM/NEAM gels without AuNRs) are measured in a similar way.

### Simulation of heat dissipation in response to NIR laser

The heat distribution, generated by the AuNRs in response to the incident light, is simulated using the FREE FEM + + (v. 3.610001) software^52^. In this simplified model, the assumption is made that heat transfer is dominated by heat conduction, and that convection and radiation are negligible. The heat equation for the simulated volume of the hydrogel film and media is solved using the finite element method (Supplementary Note 5 for details).

### Cell culture and actuation

Cell culture experiments are performed with L929 mouse-derived fibroblasts (Deutsche Sammlung von Mikroorganismen und Zellenkulturen GmbH, DSMZ ACC-2). Fibroblasts are cultured in RPMI medium with 10% FBS and 1% antibiotics/antimycotics at 37 °C, 5% CO_2_ and 95% humidity. The gel surface is modified with fibronectin or collagen I using sulfo-SANPAH as a bi-functional crosslinker (Supplementary Method 4).

Cells are seeded on the fibronectin functionalised gels at a seeding density of ~30,000 cells/ gel. After allowing cells to adhere for 8–24 h, the gels are actuated for different periods between 1 and 22 h. The gels are placed in a custom-made cell chamber and at least three gels are studied per condition (Supplementary Fig. 10).

### Analysis of cell motility

Time-lapse microscopy at 1 h intervals is performed to monitor the effect of gel actuation on cell motility using Cell Tracker^53^ software to analyse at least 44 cells per condition. Cells that fulfil the following criteria are randomly selected:

- Cells do not divide during imaging;
- Cells are not positioned at the borders of the field of view of the image (as they tend to go out of the field of view over time).

Afterwards, cell nuclei are assigned to an x–y coordinate system and tracked using the semi-automatic mode and template matching modality. Once all trajectories are obtained and examined, different parameters, such as end-to-end distance, contour length, persistence length, projected contour length and cell migration rate are computed (Supplementary Method 5, Supplementary Note 7).

### Microscopy

Laser scanning confocal microscopy is performed with a Leica SP 8 Tandem Confocal microscope using a white light laser for excitation. Suitable excitation and emission filters are applied. All imaging involving cells are performed at 37 °C to avoid excessive swelling of the hydrogels. The thickness of the hydrogels at different temperatures is determined by acquiring images in the z-stacking mode. Sequential scanning is performed to avoid cross-talk between channels. Super resolution confocal images are acquired using the stimulated emission depletion (STED) technique. A pulsed laser at 775 nm is used for depleting the fluorescence excitation. The point spread function is regulated using an in plane (xy) and a z component of the de-excitation laser. The detector is set in counting mode while imaging Elf, Hsp 70 and fibronectin. Image deconvolution is performed with Huygens Professional software (Scientific Volume Imaging B.V.) Confocal micrographs of immunostained cells are analysed using ImageJ (Fiji, version 1.52b) to quantify the nuclear MRTFA (%), focal adhesions, fibronectin secreted by cells and the levels of Hsp 70 and Elf in the cells (Supplementary Method 6). For each condition, *N* ≥ 60 cells are pooled from three independent experiments and analysed, unless otherwise mentioned.

### Mechanical properties

The 60/40 NIPAM/NEAM gels are subjected to monotonic compression at different temperatures to investigate the change in elastic moduli of the bulk gel below and above the VPTT. For this purpose, gels are prepared in circular PDMS moulds of 10 mm diameter and 1 mm height. The moulds are attached to a clean glass slide using silicon grease and 70 μL of the gel precursor solution is added to each well such that the well is filled up to the brim. A glass coverslip is placed on top of every well to obtain a flat surface and avoid evaporation and the precursor solution is subjected to UV polymerisation as described earlier. After curing, the gels are washed overnight with DI water and equilibrated with cell culture media at the required temperature overnight. An immersion chamber of the dynamic mechanical analyser (TA instruments) is used to completely submerge the sample in cell culture medium during the measurements. A pre-load force of 0.001 N and a cross-head speed of 0.05 N/min is applied to compress the gels until a force of 0.5 N is reached. At least three different samples are evaluated at each temperature. The stress is determined using the surface area of the gels, determined via the swelling measurements performed at different temperatures (Supplementary Method 7). The stress-strain curves are analysed and the bulk compressive modulus of the gels is determined as the initial slope of the curve up to 5% strain.

The local mechanical properties of the hydrogels are determined by the Young modulus of the gels via atomic force microscopy (Bruker Dimension Icon AFM, colloidal probe with an A-shaped cantilever, CP-PNPS-B from Nano and More). The probe is a silica bead with a radius of ~3.31 µm and spring constant of ~0.21 N/m, determined by thermal calibration. Samples are indented using a maximal force (relative set point) of 2 nN at three different temperatures (27, 37 and 45 °C) on a 5 × 5 raster pattern with 100 nm step size. At least 4 different areas are measured in this method to screen at least 100 points per condition. The elastic modulus, E, is obtained from the force-indentation curves using a Hertz model with the contact point as free parameter. The Poisson ratio is assumed to be 0.5.

### Statistical analysis

Statistical analyses are performed using IBM SPSS Statistics 20 software. Values are presented as mean ± standard deviation, unless otherwise mentioned. Error bars represent standard deviation. Data are analysed with a one way ANOVA and post-hoc Tukey test when homogeneity of variances (determined by Levene’s test) is valid and a Welch test with post-hoc Games Howell test when homogeneity of variances is not obeyed with statistical significance defined at *p* < 0.05, 0.01 and 0.001, represented by *, ** and *** respectively.

### Reporting summary

Further information on research design is available in the Nature Research Reporting Summary linked to this article.

## Supplementary information

Supplementary Information

Supplementary Movie 1

Supplementary Movie 2

Supplementary Movie 3

Supplementary Movie 4

Supplementary Movie 5

Supplementary Movie 6

Reporting Summary

## Data Availability

The authors declare that (the/all other) data supporting the findings of this study are available within the paper and its supplementary information files or from the corresponding authors upon reasonable request.
