# Supplementary material for: Cellular responses to beating hydrogels to investigate mechanotransduction
Source: Nat Commun. 2019 Sep 6;10:4027. doi: 10.1038/s41467-019-11475-4 (PMC6731269; doi:10.1038/s41467-019-11475-4)
Supplement: Supplementary file 1 — Supplementary Information [file 41467_2019_11475_MOESM1_ESM.pdf]

Supplementary information

Cellular responses to beating hydrogels to investigate mechanotransduction

Yashoda Chandorkar[1], Arturo Castro Nava[1], Sjören Schweizerhof[1], Marcel Van Dongen[1], Tamás Haraszti[1], Jens Köhler[1], Hang Zhang[1], Reinhard Windoffer[2], Ahmed Mourran[1], Martin Möller[1], Laura De Laporte,[1,3*]

[1] DWI – Leibniz-Institut für Interaktive Materialien e.V. Forckenbeckstr. 50, Aachen,52074, Germany.

[2] Institute of Molecular and Cellular Anatomy, Uniklinik, RWTH Aachen University, Aachen, 52074, Germany.

[3] ITMC- Institute of Technical and Macromolecular Chemistry, RWTH Aachen University, Aachen, 52074, Germany.

* Corresponding author

Supplementary Figures

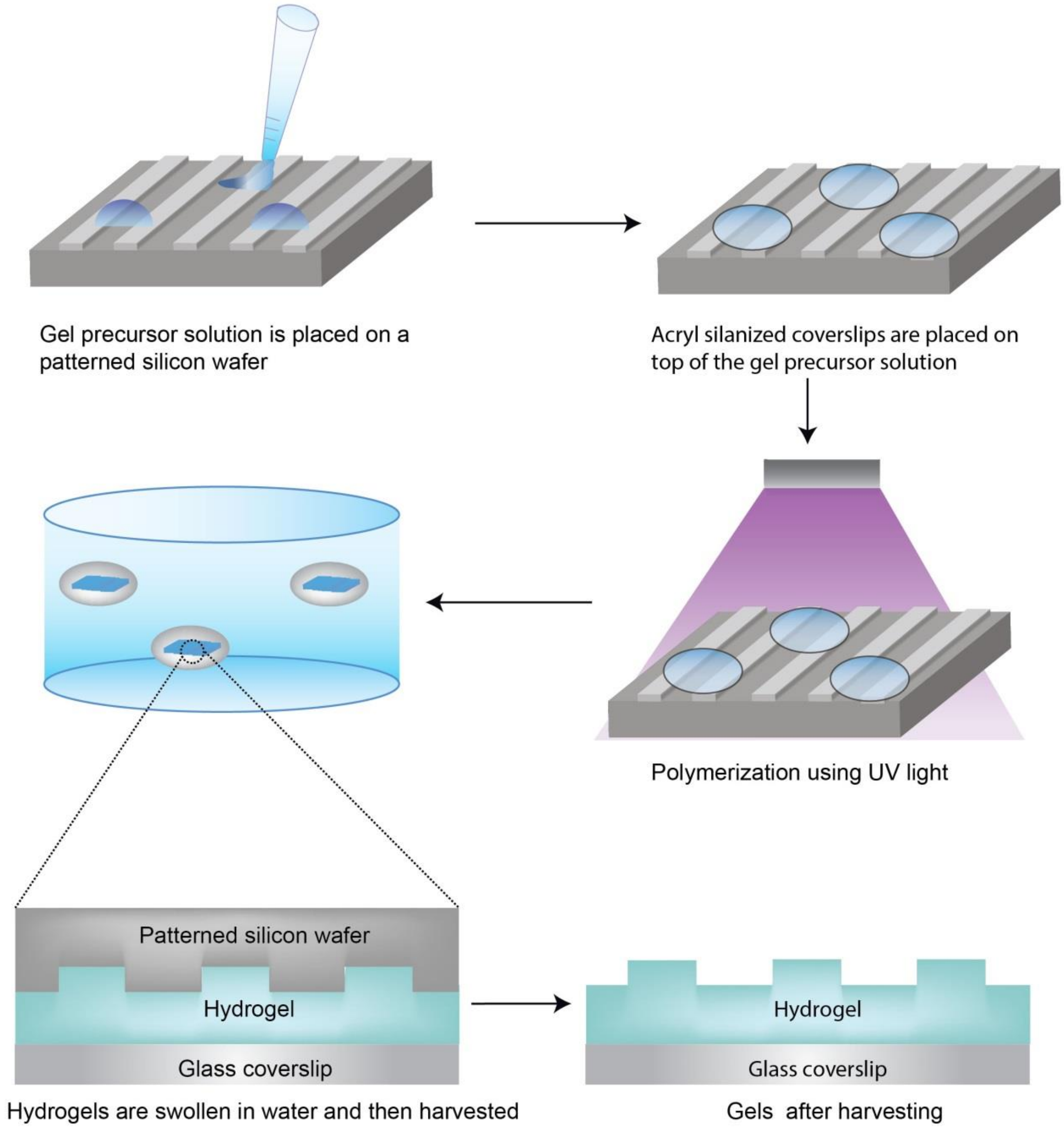

Supplementary Figure 1. Schematic showing the process of gel fabrication from the precursor solution using a silicon mould replication technique. Multiple drops of 1.5 µl gel precursor solution are placed on a silicon wafer with the desired microstructure. Acryl silanized glass coverslips are placed on top of each drop to form a thin sheet of the gel precursor solution, sandwiched between the glass coverslip and the silicon wafer. The gels are polymerized with UV light and swollen in excess deionized water overnight before harvesting.

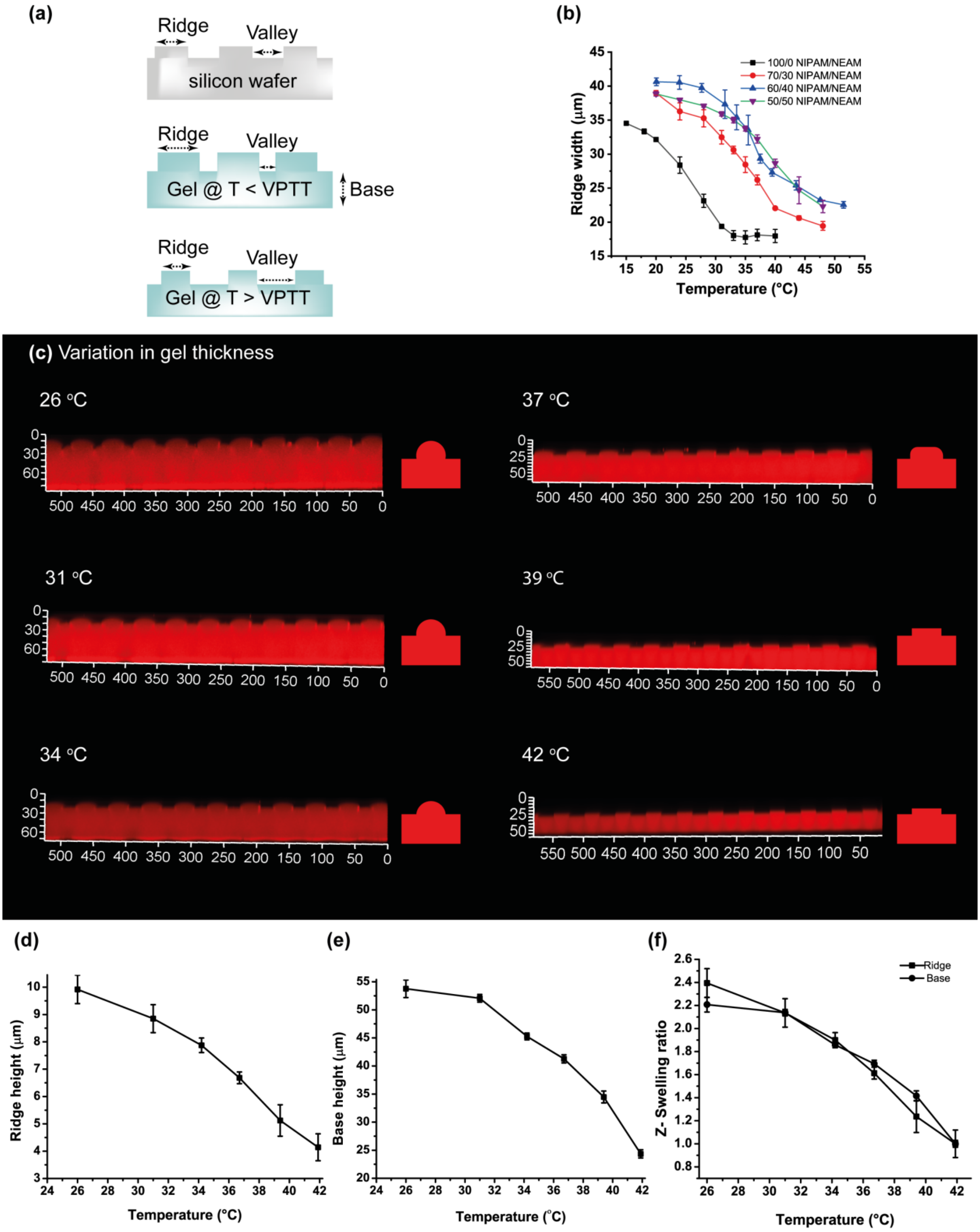

Supplementary Figure 2. (a) Schematic of the side views of the silicon wafer and the gels above and below the VPTT. The pattern on the silicon wafer has a depth of 2.7 µm. **(b)** The ridge widths at different temperatures plotted for crosslinked NIPAM/NEAM copolymers at different molar ratios (100/0 %, 70/30 %, 60/40 %, 50/50 %) to study the volume phase transition characteristics of the gel. A composition of 60/40 % NIPAM/NEAM exhibits a VPTT of ~ 37 °C and is used in this study, n = 3. **(c)** The z-stacked confocal images showing the thickness of the gel at 26, 31, 34, 37, 39, and 42 °C. The hydrogel is confined as it is bound to a glass coverslip. The changes in the shape of the hydrogel and ridges are depicted in the illustrations. **(d)** The ridge height and **(e)** the base height, as determined from **(c)**, vary with temperature. **(f)** The axial swelling ratios for the ridge and the base height are similar, suggesting isotropic actuation. Error bars represent standard deviation.

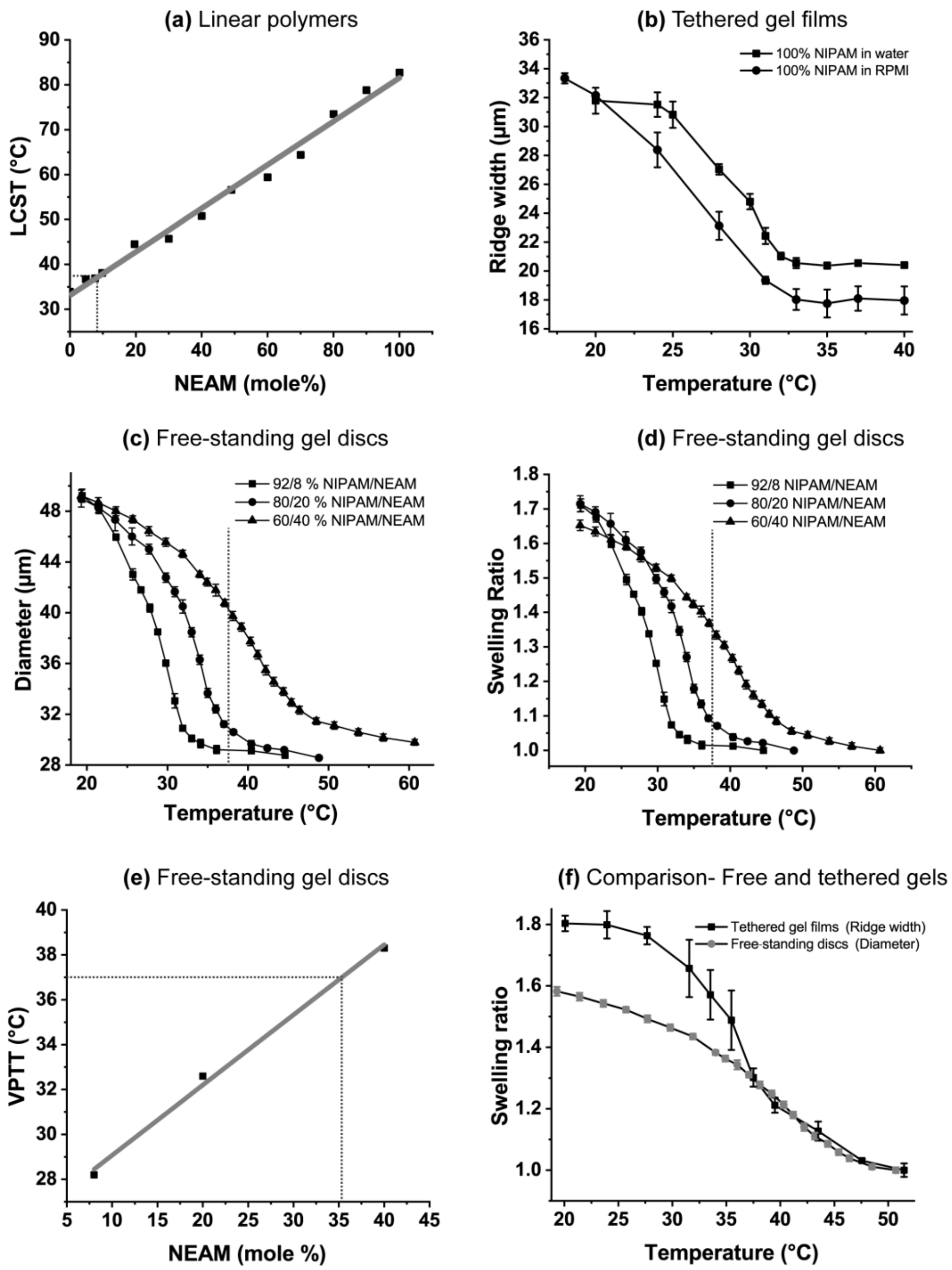

Supplementary Figure 3. (a) When linear polymers with different NIPAM/NEAM ratios are prepared using free radical polymerization and their LCST is measured in water using the cloud point method, a composition of 92/8 % NIPAM/NEAM shows an LCST of ~ 37 °C. **(b)** The VPTT of crosslinked 100 % NIPAM gels tethered to a glass coverslip (as used in the present study) is measured in water and RPMI cell culture medium to be ~ 28.8 °C and ~ 25.0 °C, respectively, demonstrating a significant decrease upon crosslinking and in the presence of media (LCST of non-crosslinked NIPAM in water is ~ 32 °C) **(c)** Diameter of free-standing gel discs, prepared with different copolymer ratios in a mould with a diameter of 30 μm and a height of 5 μm, at different temperatures in cell culture medium. **(d)** The swelling ratio of the discs obtained from the measurements in **(c)**. **(e)** A linear dependence of the VPTT with the NEAM % is observed for the free-standing discs, where a 65/35 mole % NIPAM/NEAM shows a VPTT of ~ 36 °C. This is similar to the linear dependence obtained for tethered gels (used in the present study), where 60/40 mole % NIPAM/NEAM gels have a VPTT of ~ 36 °C, shown in Figure 1c **(f)** A comparison of the swelling ratios of tethered 60/40 NIPAM/NEAM gels films and free-standing discs, showing similar behaviour, suggesting that confinement does not affect the lateral swelling of the ridges. Error bars represent standard deviation, n = 3.

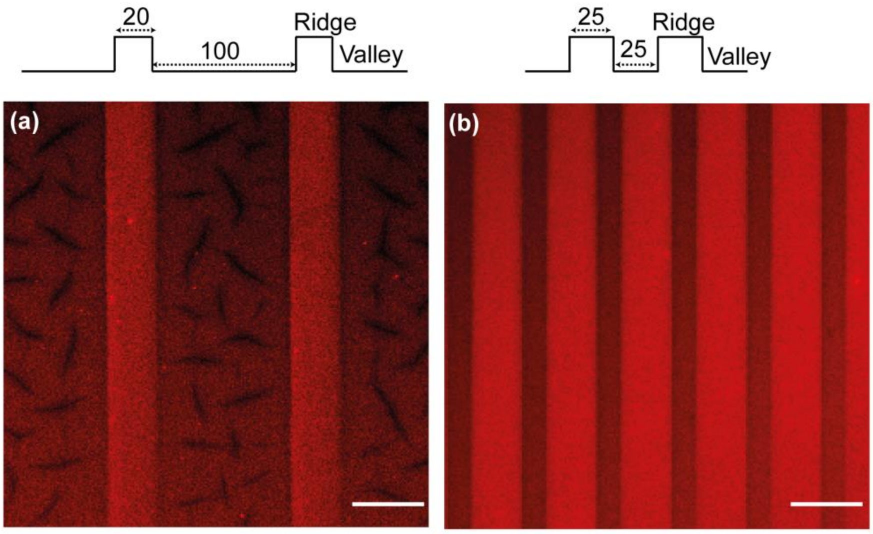

Supplementary Figure 4. Confocal images of swollen (at room temperature) 60/40 NIPAM/NEAM gels prepared with a topography of **(a)** 20 µm ridges separated by 100 µm valleys, with creases clearly visible in the valleys, and **(b)** 25 µm ridges separated by 25 µm valleys, where creases are not observed. Scale bar = 50 µm.

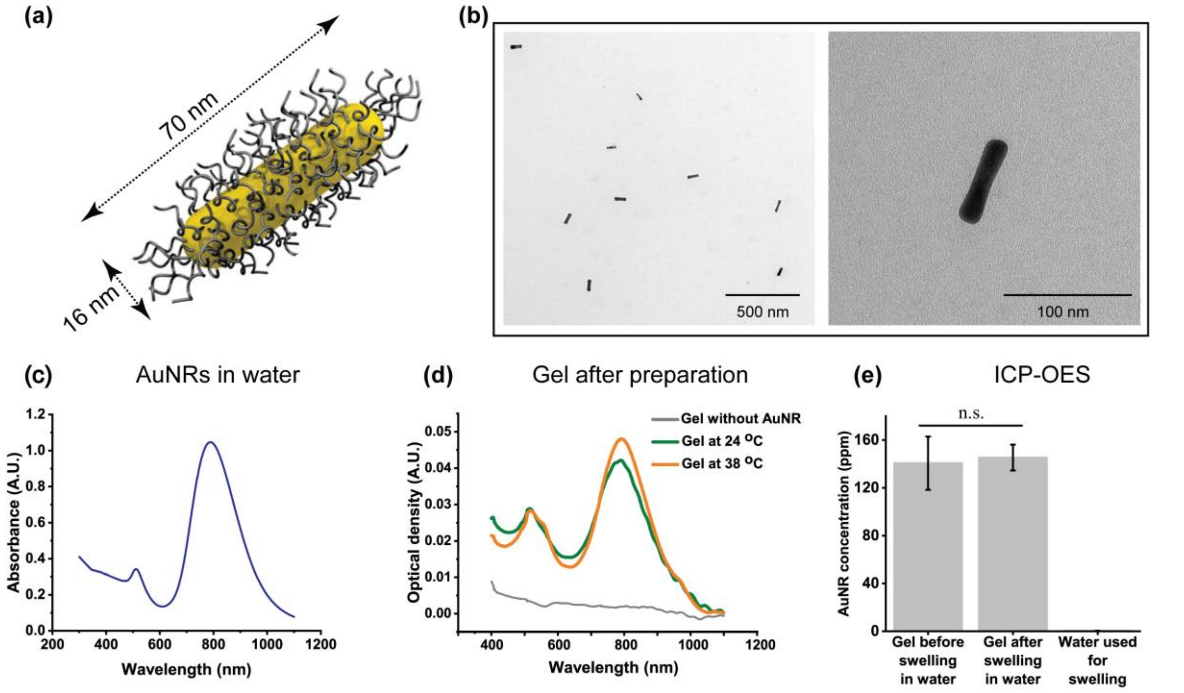

Supplementary Figure 5. (a) An illustration showing the average dimensions of the gold nanorods (AuNRs) used in the experiments, as derived from the **(b)** TEM images. **(c-d)** The UV-vis spectra of **(c)** the AuNRs dispersed in water and **(d)** the 60/40 NIPAM/NEAM gels without AuNRs and with AuNRs (gel precursor solution OD = 20) at different temperatures. **(e)** Quantification of the AuNRs using inductive coupled plasma-optical emission spectroscopy (ICP-OES) in the gel after preparation and after swelling, and in the swelling medium (water). No AuNRs are observed in the medium after 3 days, demonstrating that the AuNRs do not leach out from the gel. Error bars represent standard deviation, n = 3.

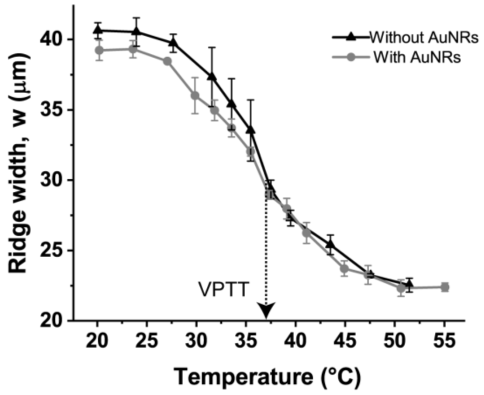

Supplementary Figure 6. Variation of the microstructure ridge width of 60/40 NIPAM/NEAM gels without AuNRs and with AuNRs (OD 100) in thermal equilibrium at different temperatures shows that the VPTT of the gels is not affected due to the addition of AuNRs. Error bars represent standard deviation, n = 3.

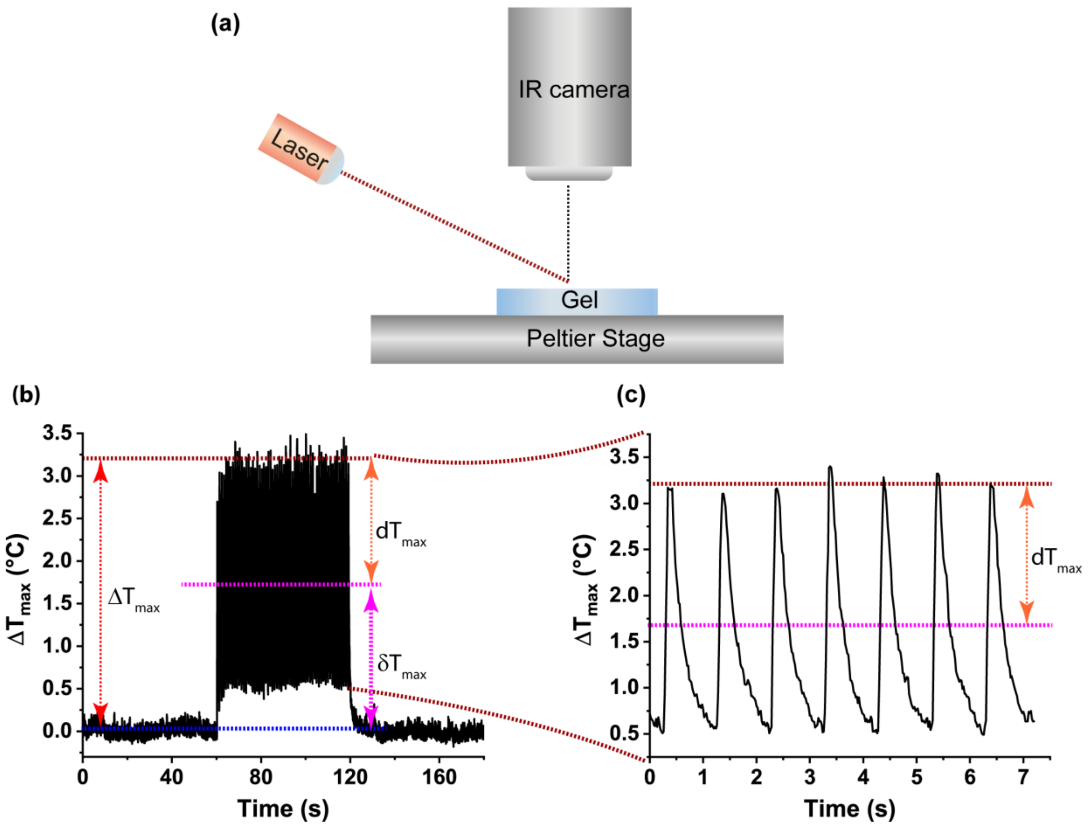

Supplementary Figure 7. **(a)** Schematic of the set-up used for measuring the gel temperature with an IR camera during photothermal heating. **(b)** The maximum temperature on the gel when the gel is stroboscopically irradiated from 60 - 120 s (340 mW, 1 Hz, 100 ms laser ON time). **(c)** The maximum temperature modulates with the pulse duration of the incident laser.

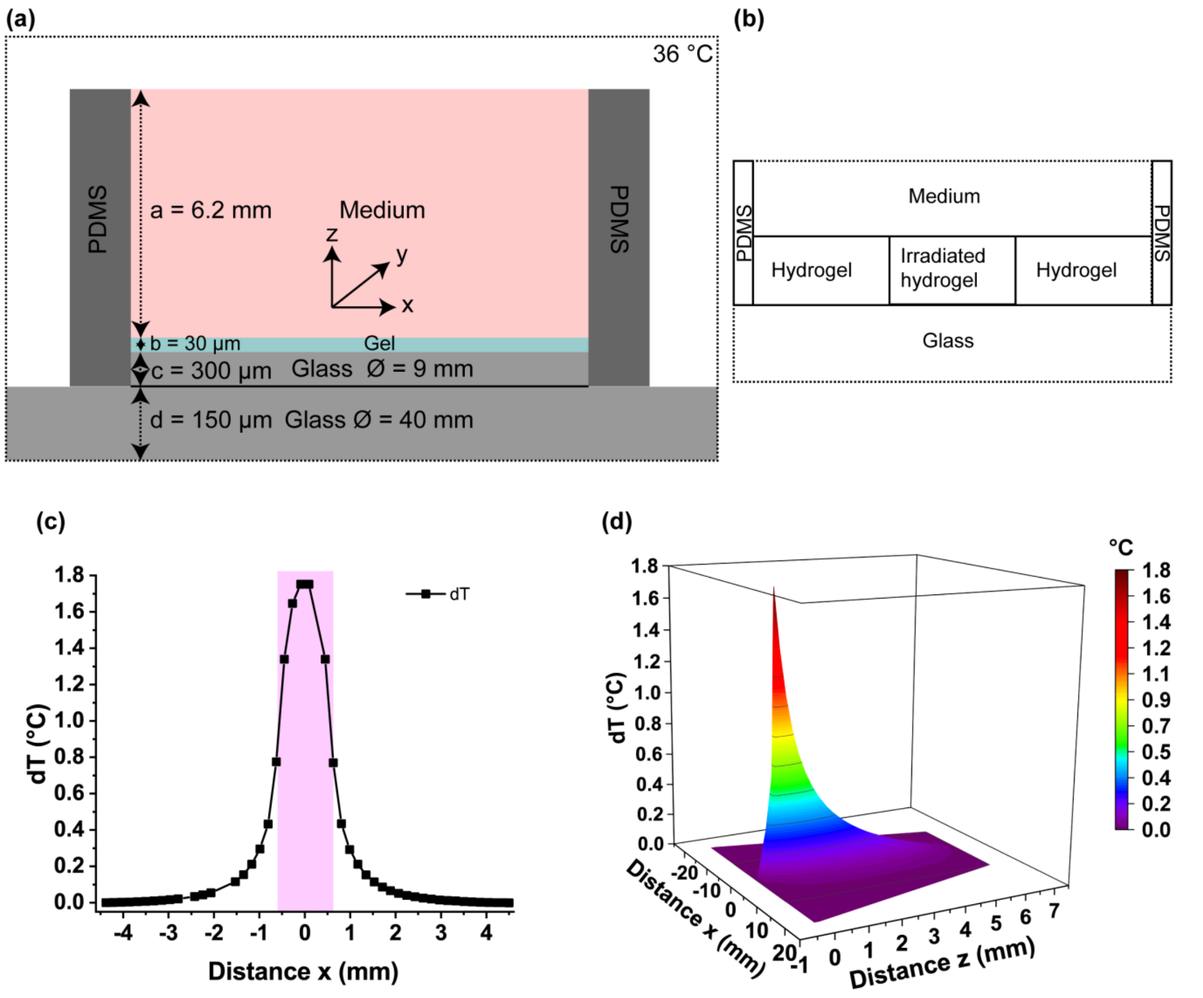

Supplementary Figure 8. (a) A schematic showing the dimensions of the setup that involves irradiation of the gel with NIR light. **(b)** A simplified version of **(a)** used for the purpose of the Finite Element Simulation. **(c)** Results of the simulation obtained for a time independent photothermal equilibrium that is achieved in response to a laser pulse of 340 mW, 100 ms laser ON time and 1 Hz frequency, depicting the variation of temperature at the surface of the gel. The laser spot is shown with the overlaid pink rectangle **(d)** A 3D distribution of the heat dissipation from the irradiated region of the gel. The asymmetric nature of the distribution in the z-direction stems from the difference in thermal conductivities of glass and medium (i.e. water).

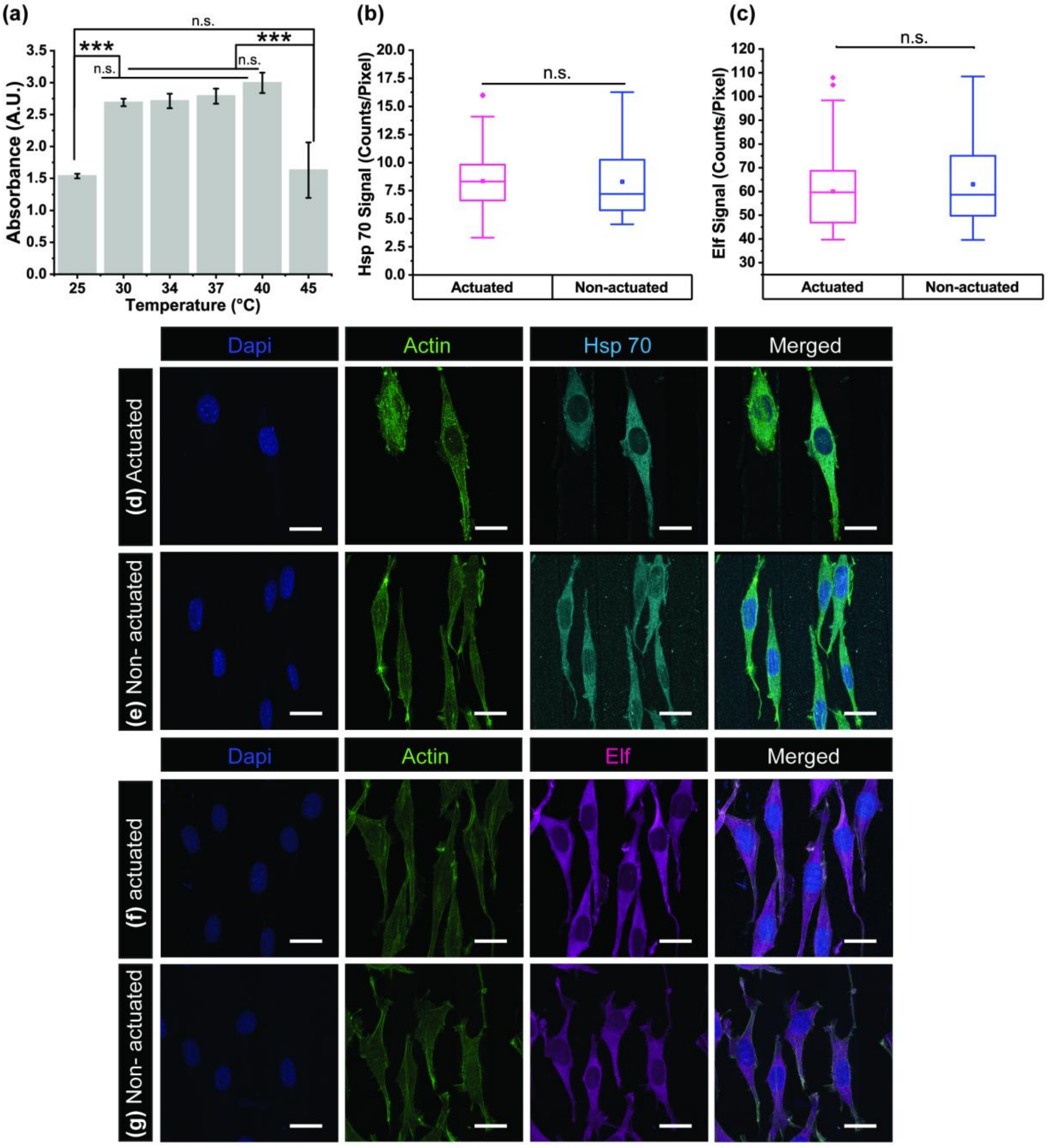

Supplementary Figure 9. **(a)** A representative MTS proliferation assay for L929 cells grown on TCPS after 24 h at different temperatures, n = 3. **(b,c)** The signal intensity of **(b)** heat shock protein Hsp 70 (n = 2, N ≥ 93 cells) and **(c)** Elf (that stains against stress granules and P-bodies, n = 2, N ≥ 155 cells) is shown in the box plots for actuated and non-actuated cells. **(d,e)** Representative images of cells stained against Hsp 70 **(d)** when actuated and **(e)** non-actuated, **(f,g)** against Elf that stains stress granules and P bodies for **(f)** actuated cells and **(g)** non-actuated cells. The non-actuation images are acquired from cells grown on the non-irradiated portion of the gel. In the box plots, the interquartile range (IQR) between the first and the third quartiles is indicated by the box, while whiskers denote 1.5 IQR. The hollow square, the horizontal line, and the filled dots represent the average, the median, and the outliers, respectively. Error bars represent standard deviation. *, **, *** are determined using one way ANOVA or Welch test, depending on the homogeneity of variances, and represent statistical significance at $p < 0.05$, 0.01 and 0.001, respectively. Scale bar = 20 µm.

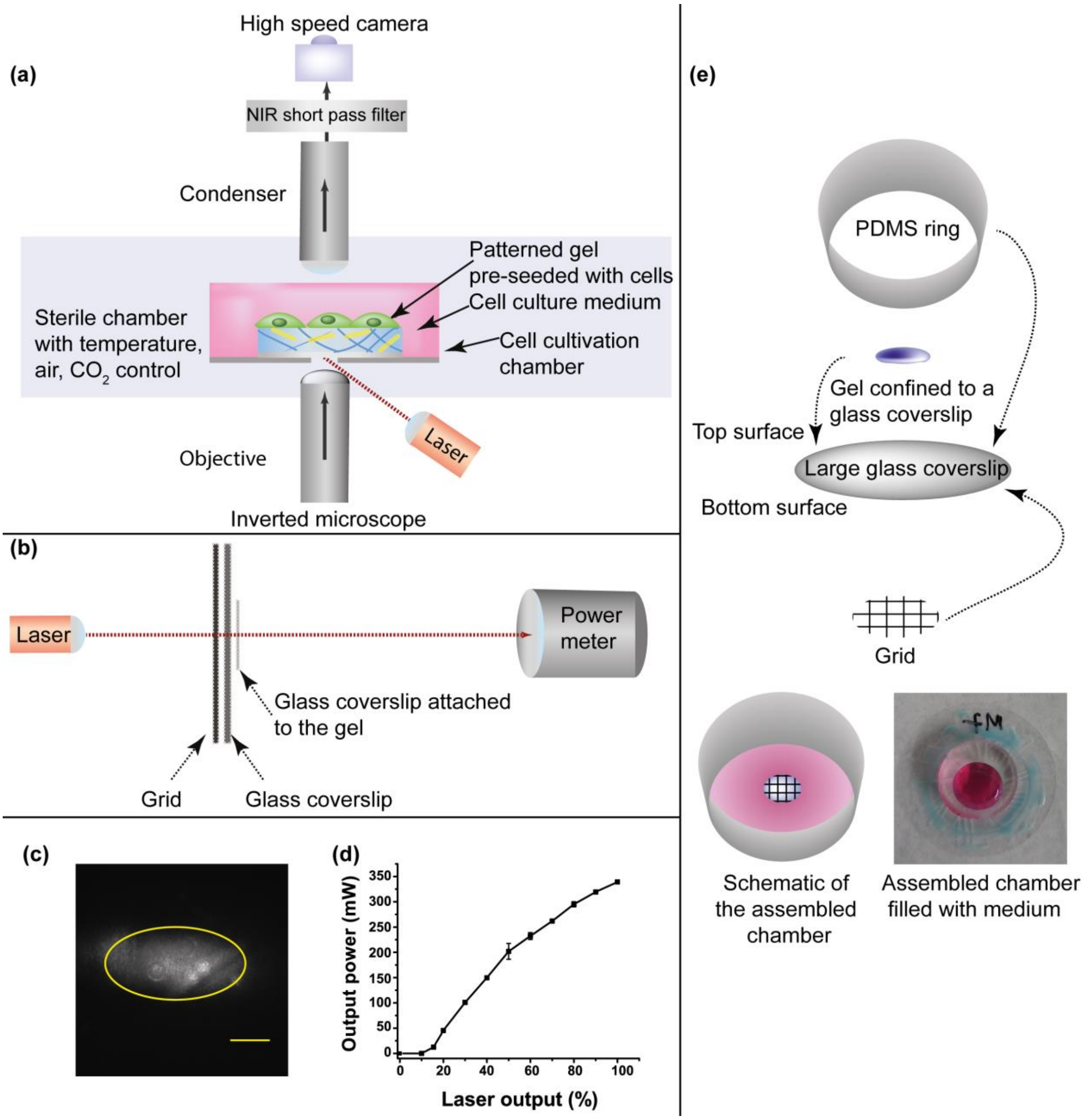

Supplementary Figure 10. (a) Schematic showing the microscope set-up, which is custom made for NIR actuation. A NIR laser is incident on the sample from the bottom. **(b)** Schematic of the set-up used to measure the laser power. **(c)** The laser footprint (340 mW) on the gel, scale bar = 500 µm. **(d)** The laser output profile, as measured with the power meter. **(e)** Schematic of the sample chamber designed for actuation. The gel, attached to a small cover glass, is glued on the sides to another larger glass coverslip and a PDMS ring is put around it to contain the media. On the opposite side of the glass coverslip, a grid with markings is glued to keep track of the actuated area. (*illustrations not to scale). Error bars represent standard deviation, n = 3.

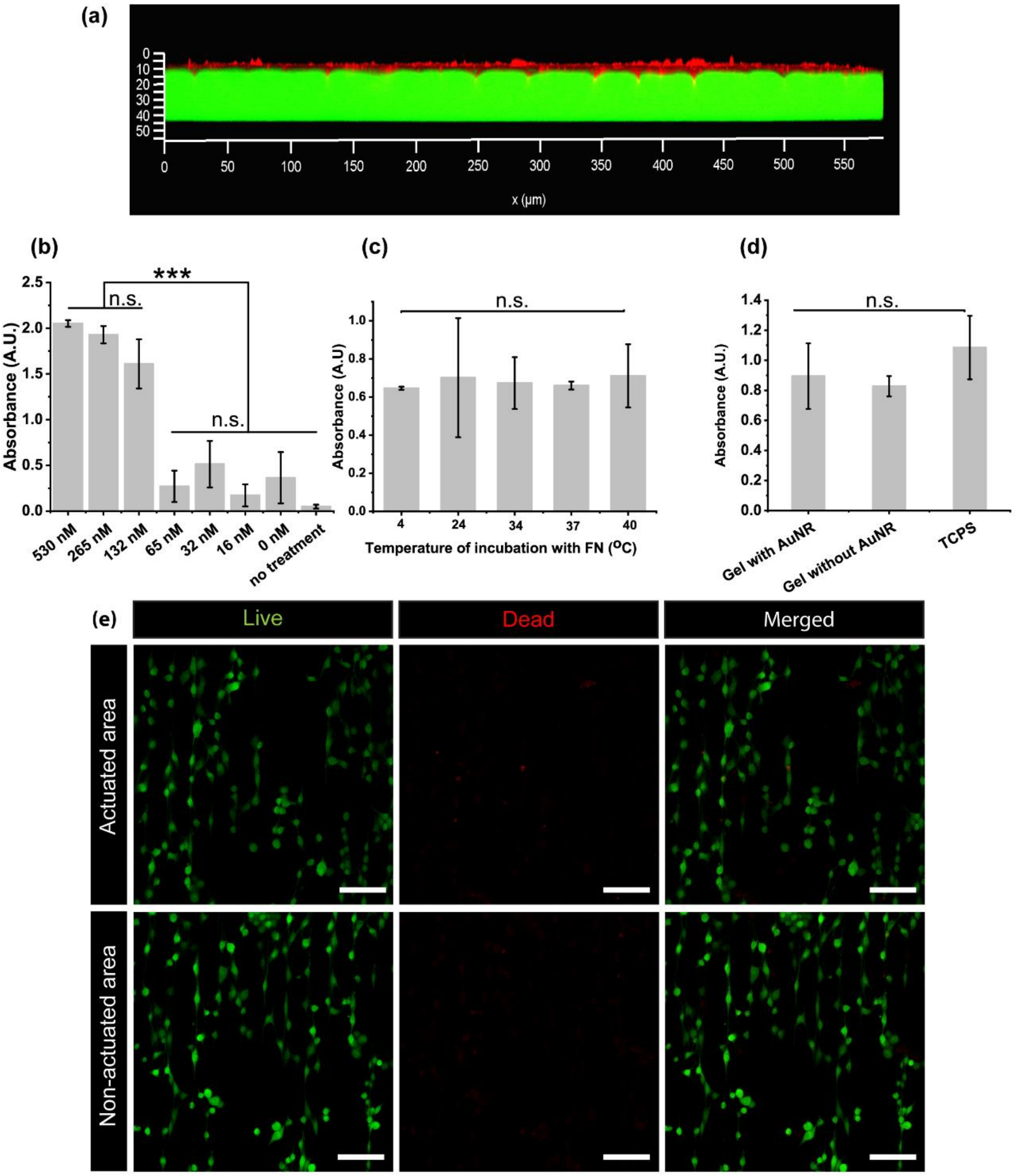

Supplementary Figure 11. (a) A confocal z-stack of a gel with fibronectin coating. Sulfo-SANPAH is used as a bi-functional crosslinker to covalently link fibronectin to the gel. Fibronectin is stained red, while the polymer is stained green. **(b-c)** The effect of the **(b)** fibronectin concentration and **(c)** temperature of fibronectin incubation on L929 cell proliferation. Based on these results, a fibronectin concentration of 265 nM, incubated at 34 °C, is further used for this study. **(d)** When gels with an AuNR concentration of 0.004 vol % are prepared, the MTS assay does not show a toxic effect as AuNRs do not leach out of the gels. **(e)** Cells seeded on the gels are exposed to a pulsed laser (340 mW, 1 Hz, 100 ms laser ON time) for a period of 22 h. No significant cell death is observed in both the actuated and non-actuated regions of the gel. Scale bar = 100 µm. Error bars represent standard deviation, n = 3.

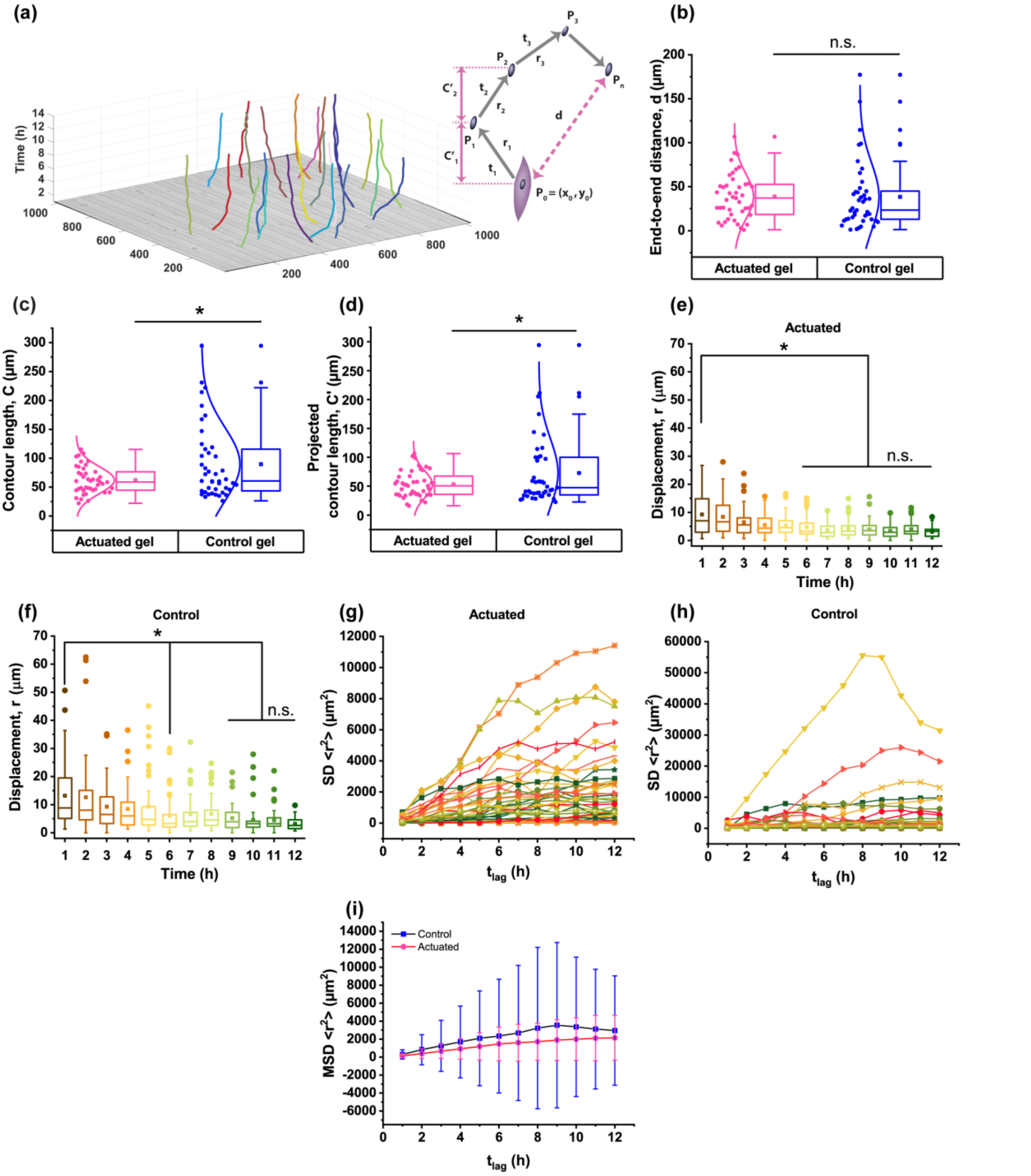

Supplementary Figure 12. (a) The trajectories of the individual cells that are traced over 12 h and a schematic showing the parameters used to quantify cell motility. **(b-d)** Box plots showing cell motility parameters on 60/40 NIPAM/NEAM gels as a result of gel actuation (340 mW, 1Hz, 100 ms laser ON time, 12h), in comparison to non-actuating 0/100 NIPAM/NEAM control gels **(b)** the net displacement of L929 cells (end-to-end distance), **(c)** the total distance travelled by the cell (contour length) **(d)** the distance covered by the cell in the direction of the patterns (projected contour length). **(e-f)** The displacement (r) of the cells in intervals of 1 h, starting from t = 0 h for **(e)** actuated cells and **(f)** control cells, showing that cells slow down and cover less distances as time progresses. **(g,h)** The square displacements (SD) for all cells plotted for each lag times **(g)** for actuated cells and **(h)** for control cells. These values are averaged to get the mean of the SD (MSD) at each lag time, which is used to determine that cells undergo erratic motion (Figure 3 c). **(i)** The MSD of all the cells is plotted along with the standard deviation. The box plots show the result for each cell (n = 2, N ≥ 43 cells). In the box plots, the interquartile range (IQR) between the first and the third quartiles is indicated by the box, while whiskers denote 1.5 IQR. The hollow square, the horizontal line, and the filled dots represent the average, the median, and the outliers, respectively. On the left of the box plot, all data points are shown, the normal distribution curve serves to guide the eye. *, **, *** are determined using one way ANOVA or Welch test, depending on the homogeneity of variances, and represent statistical significance at $p < 0.05$, 0.01 and 0.001, respectively.

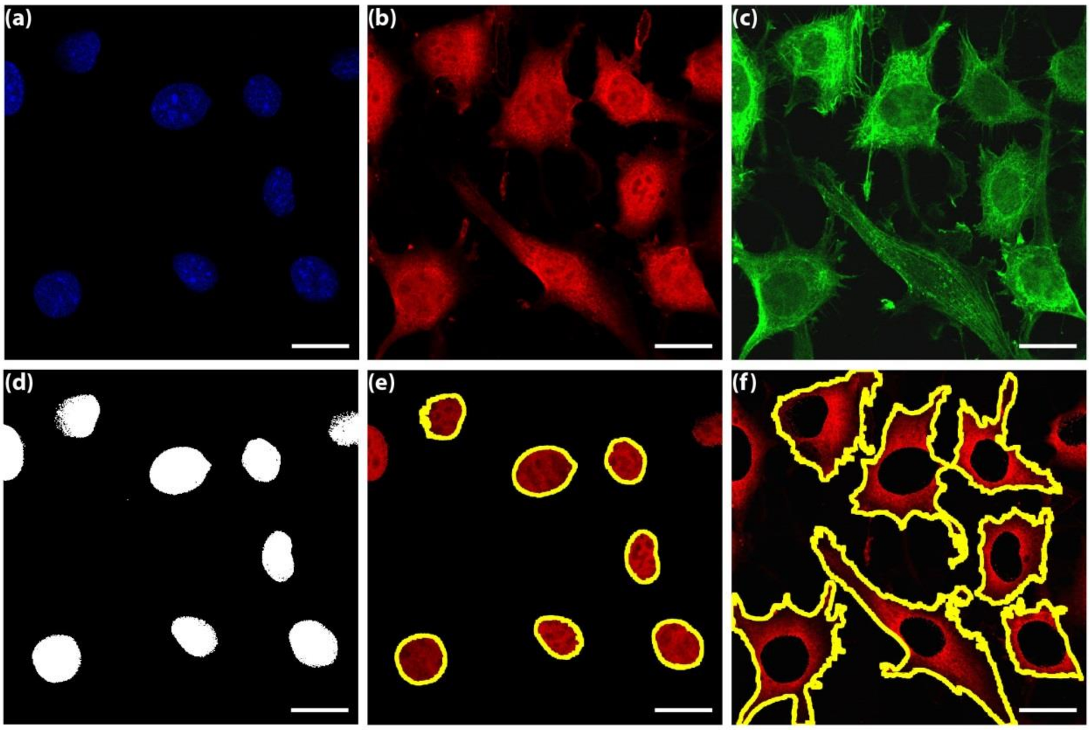

Supplementary Figure 13. Image processing for quantification of nuclear MRTFA (%) **(a,b,c)** The nucleus, MRTFA, and actin stained images, respectively. **(d)** Thresholded image of **(a)** that is used as a mask to determine nuclear and cytoplasmic MRTFA. **(e,f)** The nuclear and cytoplasmic distribution of MRTFA obtained after operating the boolean operators AND and Subtract on **(b)** and **(d)**, respectively. Scale bar = 20 μm.

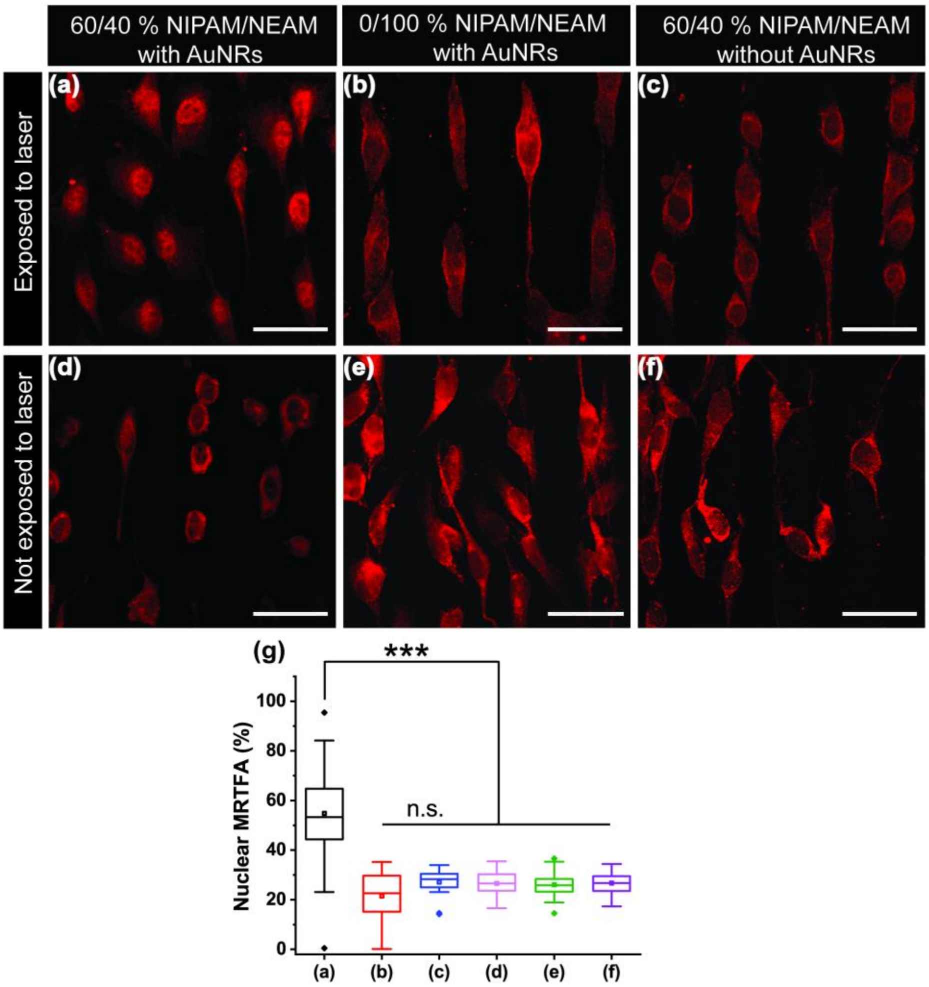

Supplementary Figure 14. Immunofluorescent staining for MRTFA in cells grown on **(a,d)** 60/40 NIPAM/NEAM gels with AuNRs which show photothermal heating with mechanical deformation (actuation), **(b,e)** 0/100 NIPAM/NEAM gels with AuNRs, which show photothermal heating without mechanical deformation, **(c,f)** and 60/40 NIPAM/NEAM gels without AuNRs, which do not display photothermal heating or mechanical deformation. The top row shows the region of the gels that is pulsed with NIR light for 12 h (340 mW power, 1 Hz, 100 ms laser ON time), while the bottom row shows a region of the gel that is not exposed to NIR light. The actuated region demonstrates translocation of MRTFA from the cytoplasm to the nucleus, while this is neither observed on the control gels nor in the regions of the gel that do not actuate. Scale bar = 50 µm. **(g)** The nuclear MRTFA (%) is measured and represented by box plots where the interquartile range (IQR) between the first and the third quartiles is indicated by the box, while whiskers denote 1.5 IQR. The hollow square, the horizontal line, and the filled dots represent the average, the median, and the outliers, respectively. On the left of the box plot, all data points are shown, the normal distribution curve serves to guide the eye. ($n \geq 2$, $N \geq 18$ cells). *, **, *** are determined using one way ANOVA or Welch test, depending on the homogeneity of variances, and represent statistical significance at $p < 0.05$, 0.01 and 0.001, respectively.

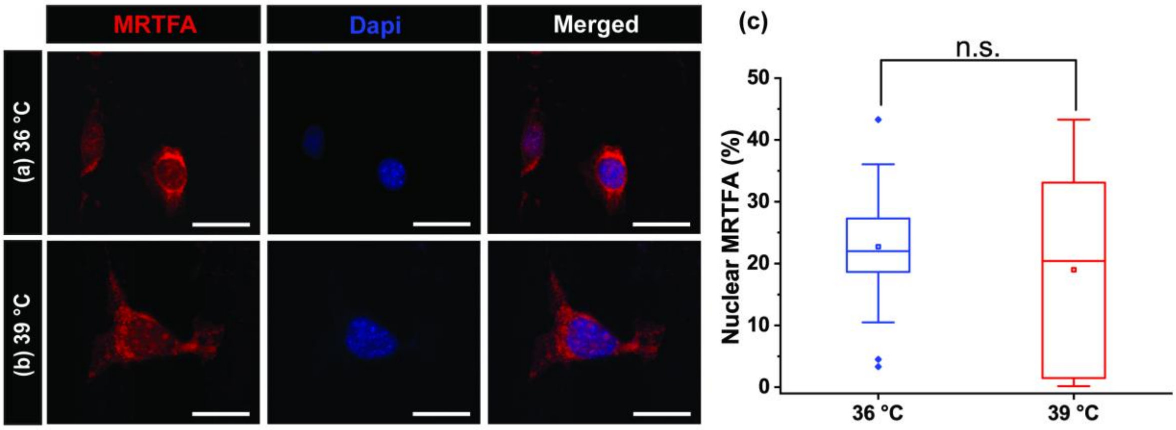

Supplementary Figure 15. Immunofluorescent staining for MRTFA in cells grown on 60/40 NIPAM/NEAM gels with AuNRs at **(a)** 36 °C and **(b)** 39 °C, where MRTFA is located in the cytoplasm at both temperatures. Scale bar = 20 µm. **(c)** Nuclear MRTFA (%) is measured and represented by box plots where the interquartile range (IQR) between the first and the third quartiles is indicated by the box, while whiskers denote 1.5 IQR. The hollow square, the horizontal line, and the filled dots represent the average, the median, and the outliers, respectively (n = 3, N ≥ 16 cells). *, **, *** are determined using one way ANOVA or Welch test, depending on the homogeneity of variances, and represent statistical significance at $p < 0.05$, 0.01 and 0.001, respectively.

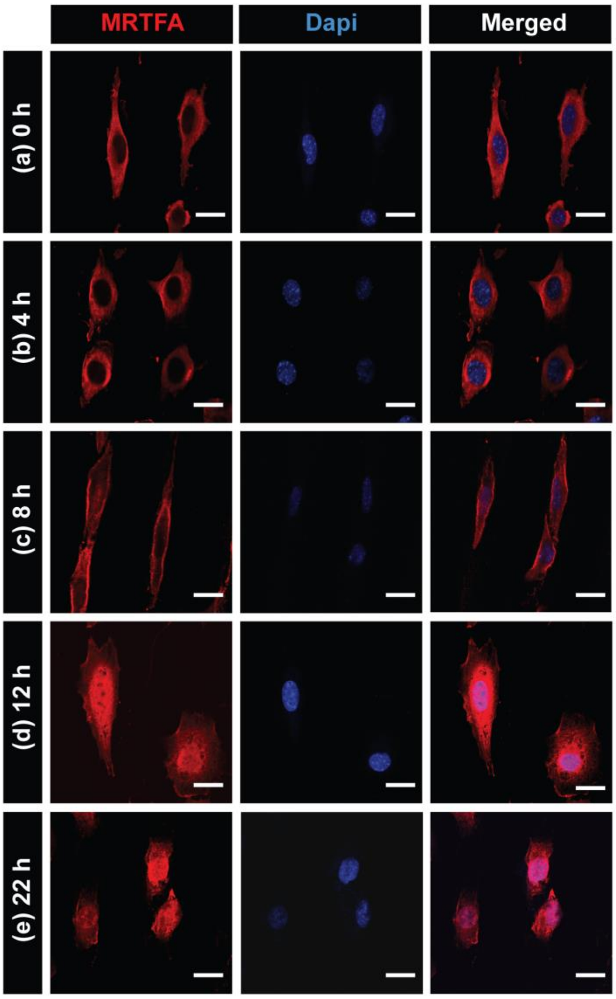

Supplementary Figure 16. Representative immunofluorescent images of cells that are present in the actuating area of the gel stained against MRTFA and Dapi (340 mW power, 1 Hz, 100 ms laser ON time) for **(a)** 0, **(b)** 4, **(c)** 8, **(d)** 12, and **(e)** 22 h. Scale bar = 20 µm.

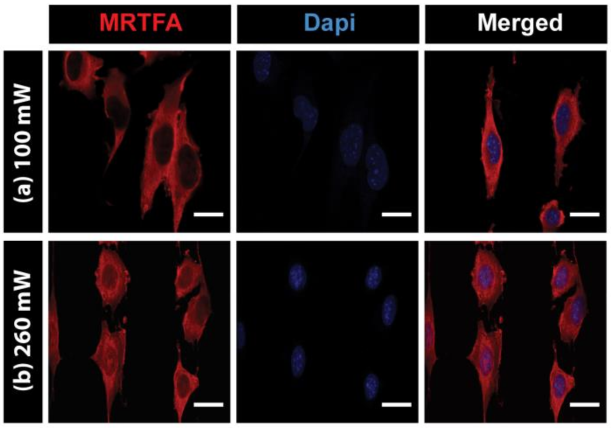

Supplementary Figure 17. Representative immunofluorescent images of cells that are present in the actuating area of the gel stained against MRTFA and Dapi (12 h, 1 Hz, 100 ms laser ON time) when the laser power is varied from **(a)** 100 mW to **(b)** 260 mW. Scale bar = 20 µm.

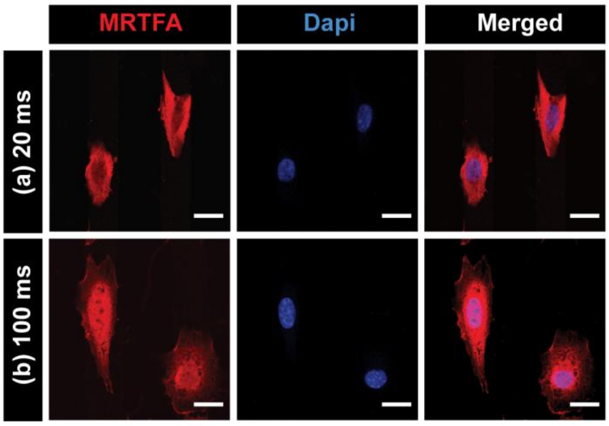

Supplementary Figure 18. Representative immunofluorescent images of cells that are present in the actuating area of the gel stained against MRTFA and Dapi (340 mW power, 1 Hz, 12 h) when the laser ON time of the pulse is varied from **(a)** 20 to **(b)** 100 ms. Scale bar = 20 µm.

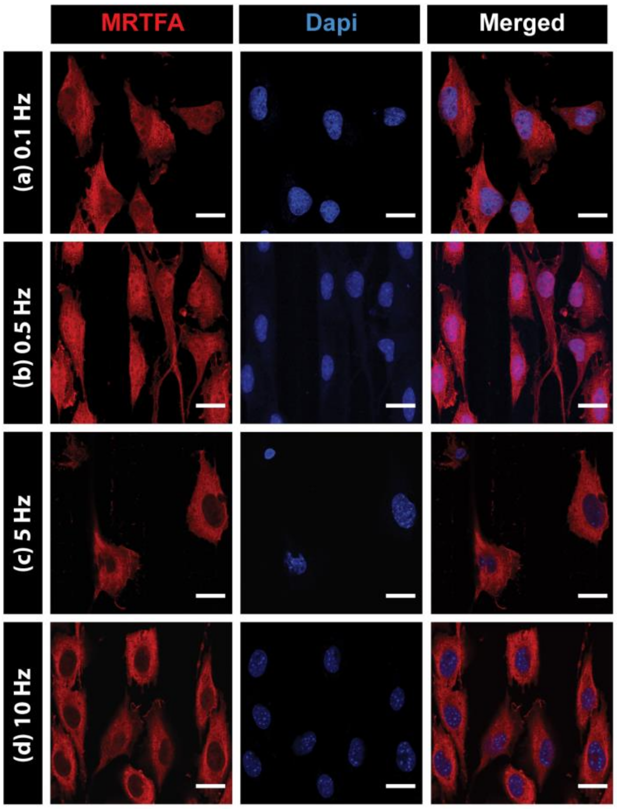

Supplementary Figure 19. Representative immunofluorescent images of cells that are present in the actuating area of the gel stained against MRTFA and Dapi (340 mW laser power, 12 h) with a pulse of 100 ms when the frequency is changed from **(a)** 0.1to **(b)** 0.5 Hz, or with a pulse of 20 ms to achieve higher frequencies of **(c)** 5 and **(d)** 10 Hz. Scale bar = 20 μm.

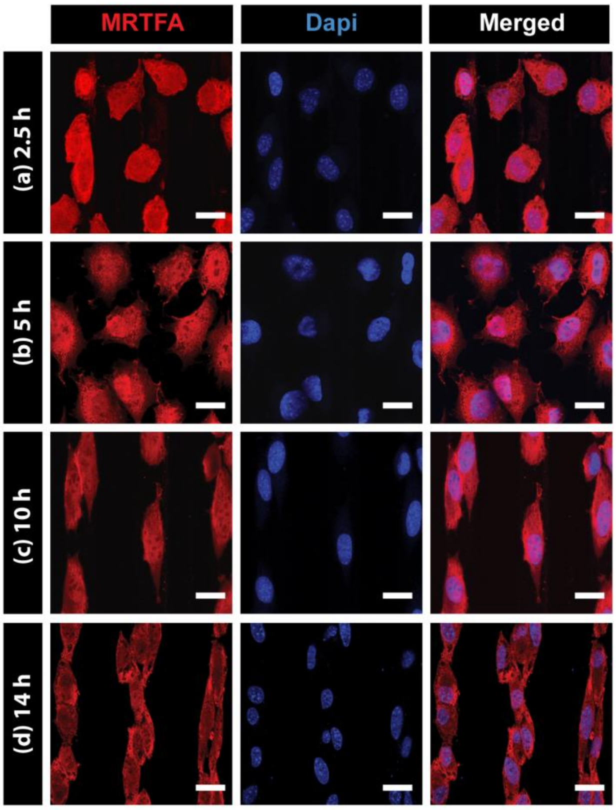

Supplementary Figure 20. Representative immunofluorescent images of cells that are present in the actuating area of the gel stained against MRTFA and Dapi (340 mW power, 1 Hz, 100 ms laser ON time, 12 h), after which the gel is relaxed for different time lengths before fixing and staining. The relaxation time is varied from **(a)** 2.5, **(b)** 5, **(c)** 10, or **(d)**14 h. Scale bar = 20 µm.

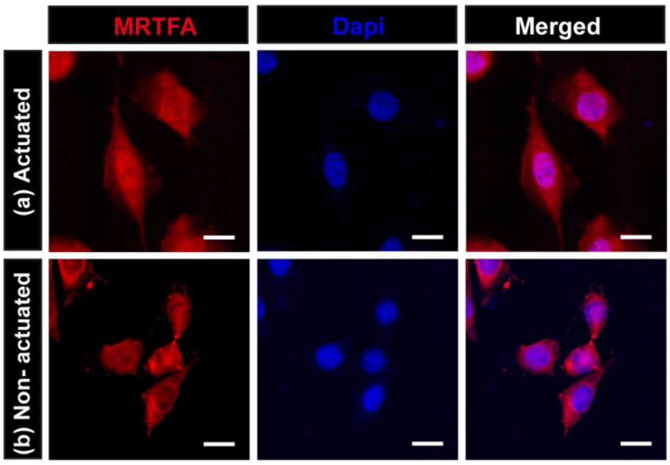

Supplementary Figure 21. Representative immunofluorescent images of cells that are present in the **(a)** actuating area (340 mW power, 1 Hz, 100 ms laser ON time) and **(b)** non-actuating area of a 60/40 % NIPAM/NEAM gel with AuNRs, functionalized with collagen I. MRTFA shuttles from the cytoplasm to the nucleus in response to 12 h actuation.

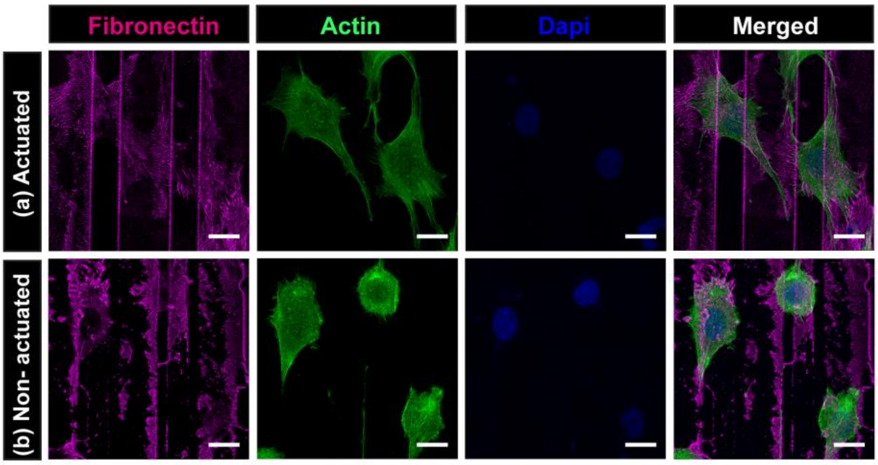

Supplementary Figure 22. Fibronectin secretion by cells grown on collagen coated gels that are **(a)** actuated for 12 h (340 mW, 1 Hz, 100 ms laser ON time) and **(b)** that are not actuated. The actuated cells also show aligned deposition of fibronectin, while a more random deposition pattern is observed for the non-actuated cells. Scale bar = 20 µm.

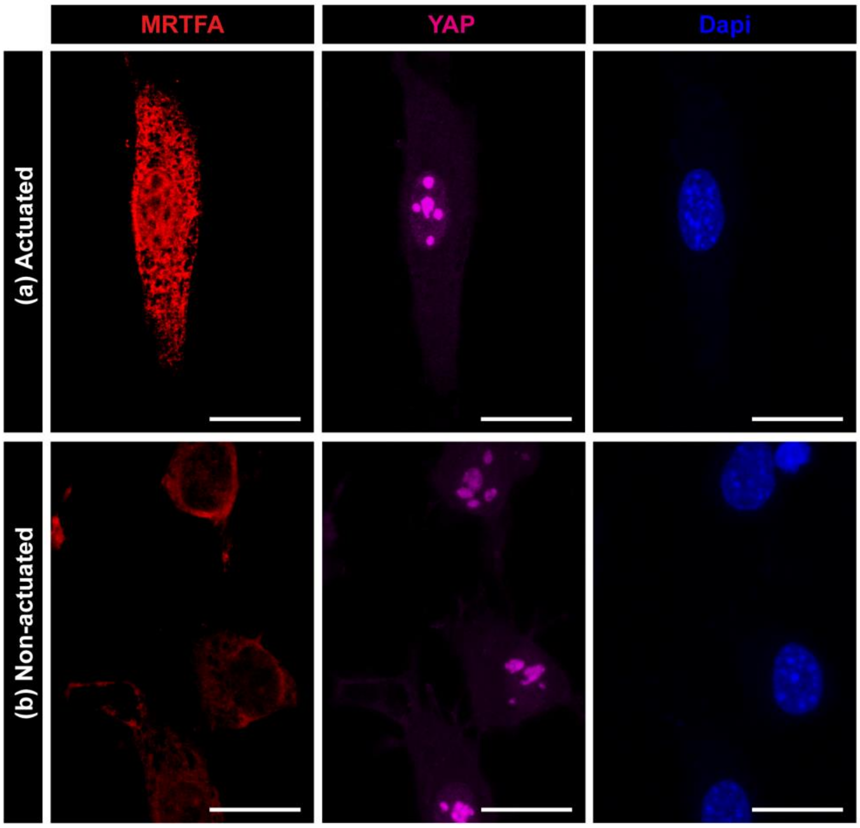

Supplementary Figure 23. Immunofluorescent images of cells that are present in the **(a)** actuating area (340 mW power, 1 Hz, 100 ms laser ON time) and **(b)** non-actuating area of a 60/40 % NIPAM/NEAM gel with AuNRs. While YAP is found in the nucleus in both cases, MRTFA shuttles from the cytoplasm to the nucleus in response to actuation. Scale bar = 20 µm.

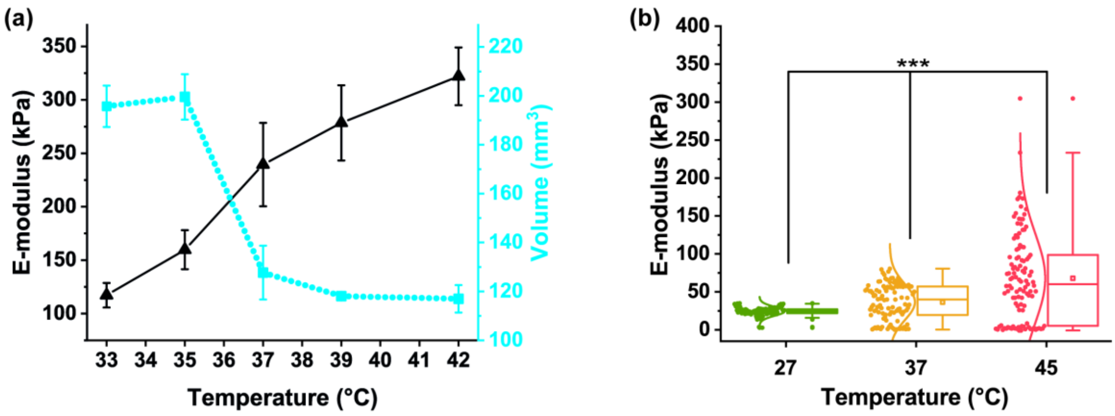

Supplementary Figure 24. **(a)** The compressive modulus of the bulk hydrogels is measured by monotonic compression at different temperatures and plotted with the volume of the hydrogels at the corresponding temperature, n = 3. **(b)** The local modulus of the confined gels tethered to a glass coverslip is measured using Atomic Force Microscopy (AFM), n = 2. The interquartile range (IQR) between the first and the third quartiles is indicated by the box, while whiskers denote 1.5 IQR. The hollow square, the horizontal line, and the filled dots represent the average, the median, and the outliers, respectively. On the left of the box plot, all data points are shown, the normal distribution curve serves to guide the eye. Error bars represent standard deviation. *, **, *** are determined using one way ANOVA or Welch test, depending on the homogeneity of variances, and represent statistical significance at $p < 0.05$, 0.01 and 0.001, respectively.

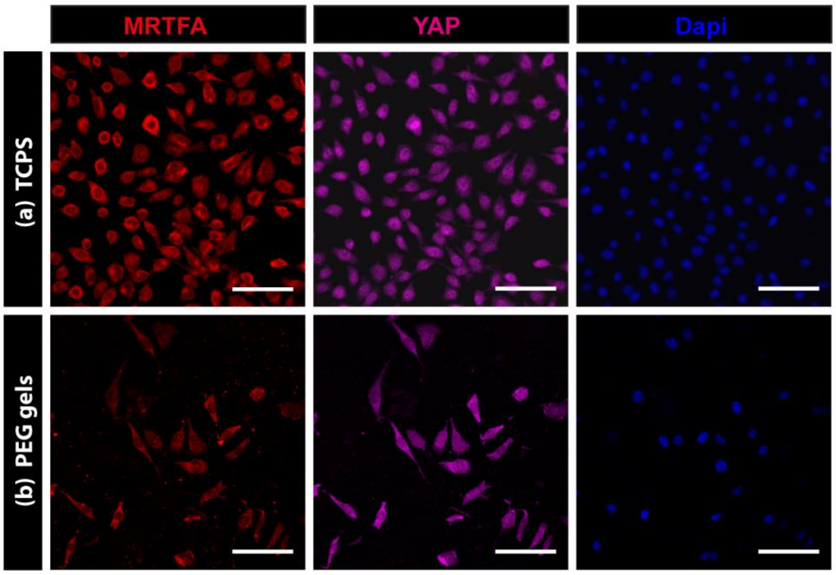

Supplementary Figure 25. MRTFA, YAP, and Dapi stained L929 fibroblast cells. **(a)** On tissue culture polystyrene, MRTFA is present in the cytoplasm, while YAP is localized in the nucleus. **(b)** On soft 1 wt % poly (ethylene glycol) gels, MRTFA and YAP are localized in the cytoplasm. Scale bar = 100 µm.

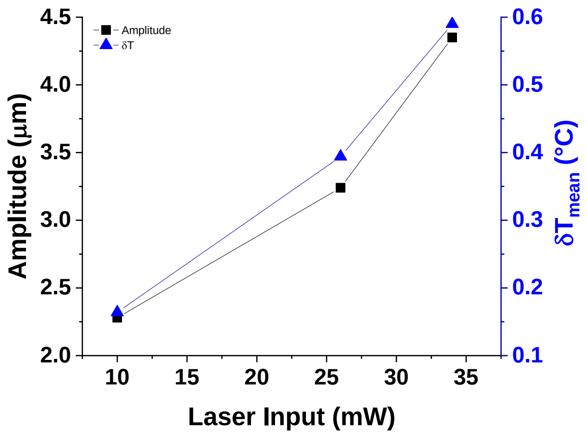

Supplementary Figure 26. A plot of the temperature changes that are experimentally measured during photothermal actuation using an IR camera and the corresponding changes in amplitude when the laser input is varied.

Supplementary Tables

Polymers/Crosslinked Gels (mole %)	Solvent	LCST/VPTT (°C)
**Linear Polymers**		
100 % NIPAM (M_n = 123,600; PDI = 1.7)	Water	~33.9
90.2/ 9.8 % NIPAM/NEAM (M_n = 154,600; PDI = 2.0)	Water	~ 38.1
60/40 NIPAM/NEAM (M_n = 95500, PDI = 2.7)	Water	~ 59.4
**Tethered gels**		
100 % NIPAM	Water	~ 28
100 % NIPAM	RPMI Cell culture medium	~ 26
60/40 % NIPAM/NEAM	RPMI Cell culture medium	~ 36
**Freestanding discs**		
92/8 % NIPAM/NEAM	RPMI Cell culture medium	~ 28
80/20 % NIPAM/NEAM	RPMI Cell culture medium	~32.6
60/40 % NIPAM/NEAM	RPMI Cell culture medium	~ 38.3

Supplementary Table 1. A summary of the LCST/ VPTT values for different compositions of NIPAM/NEAM investigated in the present study as linear polymers, tethered gel films, or freestanding gel discs in water and cell culture media.

Laser power (mW)	T ON (ms)	T OFF (ms)	Duty cycle	frequency	Energy input (mJ)	Actuation amplitude (µm)	Relative displace ment (%)	Estimated Force (nN)	Energy of actuation (pJ)
340	100	900	10	1	34	4.35	7.07	141.91	0.61
260	100	900	10	1	26	3.24	5.26	78.73	0.25
100	100	900	10	1	10	2.28	3.70	38.99	0.08
340	100	900	10	1	34	4.35	7.07	141.91	0.61
340	20	980	2	1	6.8	1.62	2.63	19.68	0.031
340	100	1900	5	0.5	17	4.35	7.07	141.91	0.617
340	100	9900	1	0.1	3.4	4.35	7.07	141.91	0.617
340	20	180	10	5	34	1.62	2.63	19.68	0.031
340	20	80	20	10	68	1.62	2.63	19.68	0.031

Supplementary Table 2. A summary of the different stroboscopic conditions for laser pulsing used in the present study, the amplitude of actuation, the calculated relative displacement, the generated forces as a result of actuation, and the energy of actuation per actuation cycle i.e. per beat. Also see Supplementary Method 8.

Parameter	Value	Units
Incident power	340	mW
Absorbance of the gel	0.068	N.A.
Gel thickness	30	μm
Spot area	1246400	μm^2
Spot radius	630	μm
Duty cycle	1:10	N.A.
$k_{hydrogel}$[1]	0.624	Wm^-1K^-1
k_{glass}[2]	1.127	Wm^-1K^-1
k_{PDMS}	0.15	Wm^-1K^-1

Supplementary Table 3. Laser and material parameters used for the simulation of the gel temperature

Supplementary Methods

Supplementary Method 1. Acryl silanization of glass coverslips

Glass coverslips (diameter = 9 mm, Merienfeld) are cleaned by sonication in water, acetone, and isopropanol for 5 min each, after which they are dried with nitrogen gas. The coverslips are activated with oxygen plasma for 15 min, 200 W, and an oxygen flow rate of 50 ml/min in a plasma coater (AK 330). After plasma treatment, the coverslips are silanized by keeping them in a desiccator at a pressure of 8 x 10^{-2} mbar with 100 μl of 3-(Trimethoxysilyl) propyl acrylate silane (Sigma) for 2 h. After 2 h, the silane is removed and the desiccator is maintained at a pressure of 8 x 10^{-2} mbar for 2 h. These acrylsilanized glass coverslips are placed in the dark in an oxygen free environment until further use.

Supplementary Method 2. Dispersion and quantification of AuNRs

Since AuNRs show a surface plasmon resonance, this property is used to quantify the amount of AuNRs in the solution. The optical density of the gel precursor solution, as well as of the hydrogels, is measured on a JASCO UV-Vis spectrophotometer from 1100 to 400 nm at 1000 nm/min. Quartz cuvettes with a path length of 1 mm are used to measure the absorbance. A gel precursor solution, which does not have AuNRs, is used as a reference solution. Hydrogels without AuNRs are used as reference for measuring the spectra of the gels. The number density of the AuNRs in the solution is calculated according to the following equation:

$n = \frac{A\ ln10}{\sigma l}$,   Supplementary Equation 1

with

n= number density of the AuNRs per unit volume,

σ = extinction cross-section of the AuNRs, which is considered to be 6100 nm^2 [3,4],

l = optical path,

A= absorption (A.U.)

The gels used in the present study are prepared from a precursor solution with an O.D. ~ 95 when calculated for a path length of 10 mm, which corresponds to 3.6 AuNRs/μm^3. To measure the spectra, gels are prepared on acryl silanized glass slides and dipped in a cuvette filled with cell culture medium. The dispersion of AuNRs in the gels is visualized by transmission electron micrography (Libra 120, Zeiss). For this purpose, very thin gels loaded with AuNRs (gel precursor solution OD 1.0) are prepared on carbon coated copper grids (PLANO, CF300-CU) and imaged.

Inductive Coupled Plasma-Optical Emission Spectroscopy (ICP-OES, ICP-atom emission spectrometer Plasma 400, Perkin Elmer) is used to determine the concentration of Au in the hydrogels with a reliable quantification limit of 50 mg/L. Hydrogels are dried at 40 °C for 48 h, ionized via consecutive nitric acid (65 %) treatment and hydrochloric acid (30 %) treatment in a microwave, and sequentially used for analysis.

Supplementary Method 3. Laser parameters and sample holder for actuation

Temperature variations in the gel are dependent on the incident laser power and the AuNRs in the gel. The laser power reaching the gel is estimated using Lambert-Beer Law.

$\frac{P_{abs}}{P_0} = 1 - 10^{-A}$ Supplementary Equation 2

Where P_{abs}, P_0 and A are the power absorbed by the gel, the incident power, and the absorbance (optical density, A.U.) of the gel, respectively. A gel with thickness ~ 30 µm prepared from a precursor solution with O.D. 20 displayed an absorbance of 0.0527 at 37 °C (Supplementary Figure 5). For a laser spot size of 1.2 mm^2 and a gel thickness of 30 µm, this leads to a laser fluence (Laser power/gel volume) of 200-1030 W/cm^3,

The sample holder for actuation comprises a grid (Cell Lattice 63571), which is fixed with glue (Twin sil®, Picodent) on one side of a coverslip (φ = 40 mm), while the small glass coverslip (φ = 9 mm), to which the gel is bound, is fixed on its sides with glue on the other side of the coverslip, such that the optical path is not obstructed by the glue. A PDMS ring is fixed around the gel to accommodate cell culture medium. The grid is used to identify the precise location of the actuated portion of the sample (Supplementary Figure 10). The laser power reaching the gel for different laser outputs is measured using a power meter with a similar optical path as used for the actuation experiments (Supplementary Figure 10 b,d).

Supplementary Method 4. Functionalization of the gel surface

The gel surface is covalently functionalized with fibronectin to promote cell adhesion using Sulfo-SANPAH mediated succinimide crosslinking. This protocol is adapted from[5,6]. A stock solution of 0.1 M Sulfo-SANPAH in DMSO is prepared and stored at -80 °C. The stock is diluted to a working solution of 3.38 mM in water just before use. After sterilization of the gels with ethanol and subsequent washing with water, 20 µl of Sulfo-SANPAH is added to the surface of each gel. To ensure uniform spreading of this solution on the gel surface, a glass coverslip is placed on top. All steps involving Sulfo-SANPAH are rapidly performed in the dark (≤ 2 min) to avoid deactivation of Sulfo-SANPAH. The gel is then exposed to UV light (Konrad Benda, 8W) for 14 min. After exposure to UV light, the activated gels are thoroughly washed in excess water at least three times, and then incubated overnight with 15 µl fibronectin

at different concentrations and at different temperatures. After optimization, 265 nM fibronectin is incubated at 34 °C (Supplementary Figure 11). An incubation temperature of 34 °C is selected based on the cell morphology observed in the images (data not shown). The gels are again washed thrice with water and immersed in cell culture medium for ≥ 1 h prior to cell seeding. Immunofluorescent staining with anti-fibronectin primary antibody is used to confirm fibronectin binding to the surface of the gel. A concentration of 66 nM is used for collagen I coating.

Supplementary Method 5. Cell motility analysis

Each cell is assigned a position P_i at time t_i, where P_i is defined by Cartesian co-ordinates (x_i, y_i). The distance between two positions P_i and P_{i+1} is denoted by r_i. Time lapse images obtained at an interval of 1 h are evaluated to measure the parameters listed below.

End-to-end distance (d_n): The net distance that is covered by the cell, thus the net displacement between the final and initial position P_n of the cell.

$d_n = \Delta(P_0, P_n)$ Supplementary Equation 3

Contour length (C_n): The actual distance covered by cells. This is usually greater than the net (end-to-end) distance, as cells do not follow a straight path. The contour length at time t_n is calculated as the sum of the distances between two consecutive positions, covered by the cells along their path till time t_n.

$C_n = \sum_{i=0}^{n-1} r_i$ Supplementary Equation 4

Cell migration rate (v): The migration rate for one cell is calculated as the distance covered by the cell within a time interval divided by the time interval.

$v = \frac{C_n}{t_n - t_0}$ Supplementary Equation 5

Persistence (L): The distance that the cell covers without changing its direction. To calculate this, the net displacement of the cell from the initial to the final position is divided by the sum of the total distance travelled to reach the final position. Hence, the persistence is the end-to-end distance divided by the contour length.

$$L = \frac{r_n}{C_n}$$ Supplementary Equation 6

Projected contour length (C_i'): The actuation is directionally controlled using a micro-patterned substrate. Therefore, the topography of the gel can also influence cell motility. To calculate the movement of cells in the direction of substrate micro-patterns, the projected contour length is calculated as the distance travelled by the cells in a direction parallel to the topography.

$$C_i' = \sum_{i=0}^{n-1} |y_{i+1} - y_i|$$ Supplementary Equation 7

To interpret the trajectory plots of cells (erratic or directed), the classical method of analysis using mean square displacement (MSD, $\langle r^2 \rangle$) as a function of lag time is used. (Supplementary Note 7).

Supplementary Method 6. Quantification of MRTFA, focal adhesions, stress granules and P bodies, heat shock protein 70, and fibronectin

Image analysis is performed using ImageJ software. Z-stack images of cells are converted into orthogonal maximum intensity projections. To quantify the nuclear MRTFA (%), Otsu's thresholding method is employed to create masks for nucleus and cell area from dapi and actin stained images, respectively. Z-stack images of cells are converted into orthogonal maximum intensity projections. To quantify the nuclear MRTFA (%), Otsu's thresholding method is employed to create masks for nucleus and cell area from dapi and actin stained images, respectively. The dapi mask and Boolean operators (SUBTRACT and AND operators for the area outside the nucleus and the nucleus, respectively) are used to create maps of MRTFA signal in the nuclear and cytoplasmic domains (Supplementary Figure13). Nuclear MRTFA

(%) is calculated from the raw intensity of MRTFA fluorescence signal in the nucleus andthe cytoplasm. The fluorescence signal is assumed to be linearly proportional to the amount of MRTFA. Focal adhesions are quantified from the paxillin stained images using a trainable WEKA segmentation[7] with particle analysis, and a threshold of 0.5 μm^2 is set to remove noise. The Elf and the Hsp 70 signal is quantified per cell by determining the mean signal intensity after thresholding cells. Fibronectin signal intensity per cell is quantified by measuring the total signal intensity in a field of view (FOV) and by dividing it with the cell number obtained from dapiimages, while the mean fibronectin intensity in a FOV is determined after thresholding from the mean signal intensity.

Supplementary Method 7. Determination of swelling properties

The swelling properties of the bulk hydrogels, used for mechanical testing, are evaluated to correlate the observed mechanical properties with the volume of the gel. After fabrication of the bulk hydrogels, they are immersed in excess DMSO to remove the unreacted monomers and washed in deionized water overnight. The gels are then immersed in cell culture medium at different temperatures and allowed to swell. The dimensions of the bulk hydrogels are measured using a Vernier Callipers at each temperature and the values are used to determine the mechanical properties.

Supplementary Method 8. Theoretical estimation of forces during actuation

To estimate the order of forces that are exerted on the cells, Hooke's law is employed, assuming that the actuation involves simple harmonic oscillations, where the driving force is provided by the incident energy from the laser. Newton's second law defines force as a quantity that can indirectly be assessed by measuring other mechanical properties, such as deformation and material properties[8]. The linear restoring elastic force *(F)* of a simple harmonic oscillator is given by $F = k * x$, where x is the displacement and k is the spring constant. Here, the spring

constant, $E * x$, is derived from the elastic modulus (E = 30 kPa) of the gel, as obtained from the AFM measurements at 37 °C, and hence, the equation may be modified to $F = E * x^2$, with x defined as half the amplitude. The energy of actuation i.e. the work done per actuation (W) is defined as $W = F * x$.

Supplementary Notes

Supplementary Note 1. Gel properties

While linear co-polymers of NIPAM/NEAM prepared by free radical polymerization in dioxane show an LCST of ~ 37 °C for 92/08 mole % NIPAM/NEAM, hydrogel films tethered to glass used in the present study have a VPTT of ~ 37 °C for a 60/40 NIPAM/NEAM molar ratio in cell culture media. Similar hydrogel films prepared with 100% NIPAM possess a VPTT of ~ 27 °C in media, compared to ~ 32 °C in water (Supplementary Figure 3b). The drop in VPTT can be explained by the presence of salts and proteins in the media.[9] The VPTT of 37 °C observed for a hydrogel with 60/40 molar ratio of NIPAM/NEAM is non-intuitive as one would expect a higher VPTT based on this ratio. This may be explained by the higher sensitivity of NEAM to the presence of salts as compared to NIPAM, which is in agreement with previous observation where the VPTT of PNEAM decreased by ~ 20 °C in the presence of 1 M NaCl[10]. This drop in VPTT is higher compared to PNIPAM, for which variable values have been reported, ranging from a decrease of ~ 5 °C (PNIPAM)[11] to ~ 8-9 °C for gels[12] in the presence of 1 M NaCl. To check if the confinement of the hydrogel film influences the VPTT, free-standing discs are fabricated from a mould of diameter 30 µm and height 5 µm, using different molar ratios of NIPAM/NEAM (Supplementary Figure 3 c-e, Supplementary Table 1). By measuring their diameter at varying temperatures in media, a VPTT of ~ 38 °C is observed for free-standing (unconfined) 60/40 % NIPAM/NEAM hydrogel discs, which is slightly higher than the hydrogels tethered to a glass slide. This may be the result of confinement, as previously reported in literature[13]

In the collapsed state, the ridges (surface topography) of the hydrogel are rectangular (~ 25 µm width) and resemble the silicon wafer pattern, while in the swollen state, the ridges appear concave with ~ 32 µm width and the grooves acquire a reduced width of ~ 18 µm (Figure 1 d). The thickness of the dry gel is 3.2 ± 0.7 µm (measured at the ridge). When performing volume phase transitions by successive heating and cooling cycles, there is no shift in the VPTT and thus no hysteresis is observed (data not shown), in contrast to pNIPAM in solution[14]. Monotonic compression of free-standing hydrogel discs at different temperatures demonstrates a rise in elastic modulus from 120 kPa at 33 °C to 320 kPa at 42 °C (dynamic mechanical analyser (DMA), Supplementary Figure 24), corresponding to hydrogel shrinking above the VPTT. In the physiologically relevant temperature range of 36-39 °C, the bulk elastic modulus varies between approximately 200 and 275 kPa. For confined hydrogel films tethered to the glass coverslips, atomic force microscopy (AFM) nano-indentation estimates the elastic modulus of the gels to range from ~20 to ~30 to ~ 60 kPa for varying temperatures from 27 °C to 37 °C to 45 °C, revealing that the elastic modulus only changes by a factor of ~1.5-2 (DMA and AFM, respectively) in the temperature range of interest (36 - 40 °C).

Supplementary Note 2. Gel surface topography

When preparing and confining flat gel films of a similar composition and thickness to a rigid substrate, creases are observed. However, in the case of the microstructured gels used here, the surface density and location of creases depend on the filling fraction of ridges relative to the microstructure period.

$$\text{Ridge filling fraction} = \frac{\text{Ridge width}}{\text{Ridge width} + \text{Valley width}} \qquad \text{Supplementary Equation 8}$$

For filling fractions smaller than 0.5, creases are present in the valleys at a distance from the edge (Supplementary Figure 4). For example, creases are observed on gels prepared with 20 µm wide ridges that are separated by 100 µm valleys with a filling fraction of 0.16. For gels

with a filling fraction of 0.5, no creases are observed, even with high-resolution confocal imaging. Based on this observation, combined with the fact that creases are depleted from the edges of the ridges, it seems that the presence of ridges suppresses crease formation. Hence, the 25 μm ridges (similar in dimension to a cell), separated by 25 μm valleys, are selected for all experiments in this report.

In addition, a larger fractional change is measured in ridge width compared to the ridge height ($\Delta h/\Delta w \sim 0.3$). This is in contrast to a confined flat film, for which $\Delta h/\Delta w \geq 1$ is expected. Together with the absence of creases when the ridge filling fraction is sufficiently high, this may indicate weak confinement of the ridges relative to the bulk of the gel below the topography.

Furthermore, the swelling ratio of the ridges is compared with the swelling ratio of free-standing discs, prepared from the same 60/40 NIPAM/NEAM composition and a diameter of 30 μm. This reveals a lateral swelling ratio of ~ 1.8 in case of the ridges, which is only marginally larger than the change in diameter of the free swelling discs (~ 1.6) (Supplementary Figure 3). This shows that although the ridges are connected to the surface of a tethered hydrogel film, their swelling and shrinking ability in response to temperature changes is not compromised.

Supplementary Note 3. Photothermal actuation

Considering the length scales involved in this photothermal hydrogel system, the AuNRs are ~ 140 times smaller in dimension than a typical cell (assumed to have a size of ~ 10 μm). Moreover, the generated heat can be controlled using a pulsed laser, which enables tunable gel actuation, depending on the kinetics of the gel's phase transition. In response to the incident laser, the AuNRs heat up the surrounding medium and gel within nanoseconds to reach a steady state[4]. The characteristic time for swelling/shrinking (τ_{charac}) in the irradiated portion of the

gel can be estimated based on the theory of kinetic swelling of gels and the collective diffusion coefficient, D_{gel}[15].

$$\tau_{charac} = \frac{\alpha^2}{D_{gel}} \qquad \text{Supplementary Equation 9}$$

with $\alpha =$ linear size of the smallest dimension. Assuming that D_{gel} for PNIPAM gels is ~ 4.0 x10^{-7} cm^2s^{-1},[16] the characteristic time for swelling/shrinking of a 60/40 NIPAM/NEAM hydrogel film with 30 µm height is estimated to be ~ 23 s at 36 °C. On the other hand, the thermal equilibration time of the gel required to reach a steady temperature due to the photothermal effect depends on the thermal diffusivity of water (k = 1.4 x 10^{-3} cm^2s^{-1}) (τ_{rel}, *rel* stands for relaxation).

$$\tau_{rel} = \frac{\alpha^2}{k} \qquad \text{Supplementary Equation 10}$$

Using Supplementary Equation 10, the typical time for the irradiated gel spot to reach an equilibration temperature is estimated to be ~ 6.4 ms. As the stroboscopic irradiation is performed with duty cycles (ON time) ranging from 20-100 ms, the thermal equilibration process is assumed to be complete. Hence, thermal confinement is avoided due to heat diffusion out of the target area into the surrounding cell culture medium and the gel swells and shrinks[4]. Thermal confinement is undesirable as it can lead to very high temperatures in the exposed area of the gel and impair actuation[17]. Due to the large heat sink, the temperature in the medium or in the non-exposed portion of the gel does not increase, leading to local actuation only in the target area. The laser pulses are selected to avoid overheating of the sample and keep the mean temperature in the actuated area always below 38.5 °C. However, since the laser ON (20 or 100 ms) and OFF time (80-900 ms) are shorter than the characteristic time required for shrinking and swelling of the gel (τ_{charac}, *23 s*), respectively, the gel is modulated around a steady state during actuation but does never fully shrinks or swells (Movie 5).

Supplementary Note 4. Estimation of gel temperature

To enable visualization of the initial contraction when the laser is turned on and the final swelling when the pulsing is stopped, the laser is pulsed between 60 and 120 s (100 ms pulses, 340 mW). Figure 2 shows that there is a spatial distribution of temperature changes, confined to the laser spot. The local total increase in temperature, ΔT_{mean}, can be subdivided in to two parts: the overall increase in the steady state temperature compared to the initial state when the laser is OFF (δT_{mean}) and the additional fluctuations in temperature when the laser is ON (dT_{mean})

$$\Delta T_{mean} = \delta T_{mean} \pm dT_{mean} \qquad \text{Supplementary Equation 11}$$

From the mean temperature measurements, the estimated values are δT_{mean} ~ 0.6 °C and dT_{mean}~ 0.3 °C leading to a net ΔT_{mean} of ~ 0.9 °C. Besides the average temperature, we also measure the maximum temperature during actuation (Supplementary Figure 7). This is the maximum temperature recorded in the gel and does not reflect the temperature of the entire gel.

$$\Delta T_{max} = \delta T_{max} \pm dT_{max} \qquad \text{Supplementary Equation 12}$$

The maximum total temperature increase ΔT_{max} in the irradiated portion of the gel is approximately 3.0 °C at equilibrium, with δT_{max} ~ 1.7 °C. The maximum temperature (due to positive oscillations) around the new equilibrium (dT_{max}) isaround ~ 1.5 °C, keeping the maximum temperature experienced by the cells below 39.0 °C and thus below the temperature of heat shock for cells[18]. Therefore, no portion of the gel is over-heated beyond the hyperthermia temperature, even after prolonged actuation. While the temperature increases very quickly leading to a rapid collapse, the recovery is slower during one actuation cycle (Movie 5).

In addition to the temperature measurements using an IR camera, the changing ridge width of the gel during actuation is measured, demonstrating a ridge width of ~ 32 μm before light

exposure and a change in ridge width from ~ 31.7 to 27.4 µm and vice-versa during actuation. Based on the calibration curve correlating the ridge width with temperature, obtained when the gels are heated without light in medium and at thermal equilibrium (Figure 1 e), the temperatures are calculated to be ~ 35.7 °C before actuation and alter between ~ 36 and 39 °C during the laser pulse (340 mW power, 1 Hz, 100 ms ON time). This measurement method is in close agreement (within experimental error) with the IR-measured temperatures, and hence, an adverse effect of temperature on cells is prevented.

Supplementary Note 5. Simulation of heat dissipation from the hydrogel film in response to a NIR laser

Local actuation of the gel is driven by local temperature changes. The temperature changes are kept at a minimum to prevent overheating of the gel, which can lead to adverse effects on cells. Infrared imaging is used to measure the temperature changes that occur in the irradiated zone on the gel. However, it is well known that time dependent measurements of the local temperature resolved in three dimensions in such small volumes are prone to error. To gain more insight into the heat dissipation during photothermal heating of this system, heat transfer is modeled using finite element modeling.

In this basic model, all time dependent variations of the system, such as volume changes and the increase in the AuNR density that occur due to the collapse of the gel are ignored. Based on the results from the infrared experiments, it is assumed that a steady state is rapidly reached during irradiation ($t <$ laser ON time). Therefore, heat diffusion and the temperature distribution across the gel surface is calculated for the situation after photothermal equilibrium is achieved. It is assumed that heat conduction dominates the scene (thus convection and radiation are neglected) and that the media and the side border of the gel are in thermal equilibrium with the environmental chamber (temperature constant at 36 ^{o}C). Another assumption is that the power

absorbed by the AuNRs is uniformly distributed over the volume of the irradiated gel, resulting in an isotropic heat flux from the irradiated volume at any time.

The actuation chamber consists of the gel that is covalently attached to a small glass coverslip (diameter = 9 mm, thickness = 300 μm), which is in turn fixed on a larger glass coverslip (diameter = 40 mm, thickness = 150 μm) with an assumed perfect contact. The hydrogel is a film (height 30 μm at 36 °C) on top of the 9 mm glass coverslip and is assumed to be dominated by water. A central 1.2 mm^2 spot is irradiated with the laser and the average heat flux is calculated based on the incoming power and the geometry of the spot. To estimate the temperature distribution, an imaginary surface is added above the gel across the heat chamber, 500 nm above the gel where cells are expected to grow, and is divided into 50 points. The topography present on the surface of the gel is neglected and the diffusion of photothermal heating from an infinite hydrogel film that is placed on a glass coverslip and immersed in water is simulated. The Fourier equation is converted into the formalism of finite element method[19] giving the following equation to solve:

$$\int_{V} -k\nabla T \nabla v dV = \oint_{A} qvdA \qquad \text{Supplementary Equation 13}$$

where v is the variation function in the mesh, vanishing at the boundary, $v(A) = 0$, T is the temperature, V is the volume, q is the heat flux incident on the sample, which is 0 everywhere outside the area of irradiation, A is the area exposed to the laser i.e. the heat source. The outer boundary of the sample is at 309.15 K (36 °C).

The material parameters used in the simulation are listed in Table 3. The gel is irradiated using laser pulses of 100 ms at 1 Hz, with a laser power of 340 mW. The absorbance value of a gel prepared from a precursor solution with OD 100 is used to calculate the power reaching the surface of the gel, P_{abs} as determined by the Lambert-Beer law (Supplementary Method 3) Using the parameters mentioned above, the heat intensity for a duty cycle of 10 % is given by

$$Q = \frac{P_{abs}}{\text{Central area}} \approx 1875\ \text{W/m}^2 \qquad \text{Supplementary Equation 14}$$

The simulations show that the average temperature of the irradiated area is ~ 1.8 °C higher than the surrounding temperature. This is in very close agreement with the IR temperature measurements revealing a ΔT_{mean} ~ 1 °C. The heat dissipation over the hydrogel in the z direction (Supplementary Figure 8 d) is asymmetric, suggesting that a major part of the heat is dissipated through glass. This finding is also significant for cells, as it conveys that cells are not exposed to elevated temperatures.

Supplementary Note 6. Surface functionalization of the gel to support cell growth

Physisorption of fibronectin to the gel does not result in a uniform coating and leads to inhomogeneous cell growth (data not shown). Therefore, Sulfo-SANPAH is used as a bi-functional crosslinker to covalently bind the primary amines of fibronectin using succinimide chemistry (Supplementary Method 4)[20]. The presence of fibronectin on the surface is confirmed via an anti-fibronectin antibody (Supplementary Figure 11) and the optimal concentration of fibronectin for the growth of L929 mouse fibroblast cells is 265 nM, as a further increase does not significantly increase cell attachment and growth after 2 days. In addition, neither the temperature (4 - 40 °C), at which fibronectin is incubated, nor the presence of AuNRs, affect adherence and proliferation of fibroblasts. The latter is not surprising as the AuNRs do not leach out into the medium.

Supplementary Note 7. Analysis of cell migration trajectories

A decrease in migration rate upon actuation is reflected in the contour length (sum of the distance covered by the cells in each step). Furthermore, the migration rate estimated by the

displacement in every hour decreases significantly over time for both cases (Supplementary Figure 12 e,f).

The effect of persistence is reflected in the contour length (distance covered by the cells) and the end-to-end distance of the trajectories (the net displacement of the cell from time t = 0 h to t = 12 h, Supplementary Figure 12 a-d).

The trajectories of single cells are analyzed by calculating the mean-squared displacement (MSD) as a function of lag time[21]. The MSD reflects the average end-to-end distance that is traversed by the cells during the lag time interval. In this study, the displacement (r) is calculated from time lapse frames acquired at time intervals (Δt) of 1 hour (13 frames acquired over 12 h, including the initial time t = 0). The squared displacement (SD) for a defined lag time equals the square of the end-to-end distance between t = 0 and t = nΔt, with n ranging between 0 and 12. The MSD $< r^2 >$ at a given lag time $n\Delta t$ is calculated as the mean of the squared displacements (SD) for all cells (N = 43 cells) using Supplementary Equation 15

$$\mathrm{MSD} < r^2 >= \frac{1}{N}\sum_{i=1}^{N}[P_{i,(n\Delta t)} - P_{i(0)}]^2 \qquad \text{Supplementary Equation 15}$$

Where $P_t\,(x, y)$ is the position vector of the cell at time t, described by the Cartesian co-ordinates (x,y). Comparing the motion to particle diffusion[22], the mean squared displacement shows the characteristics of anomalous subdiffusion. However, due to the high standard deviation of the data, no fitting is possible. The trajectories also indicate persistence related to the microstructure of the surfaces and an increase of this persistence in correlation to the mechanical actuation of the gels.

Supplementary References

1 Touloukian, Y. S., Liley, P. & Saxena, S. Thermophysical properties of matter-the tprc data series. volume 3. thermal conductivity-nonmetallic liquids and gases. (Thermophysical and electronic properties information analysis center (1970).

2 Powell, R., Ho, C. Y. & Liley, P. E. *Thermal conductivity of selected materials*. Vol. 8 (US Department of Commerce, National Bureau of Standards Washington, DC (1966).

3 Jain, P. K., Lee, K. S., El-Sayed, I. H. & El-Sayed, M. A. Calculated absorption and scattering properties of gold nanoparticles of different size, shape, and composition: applications in biological imaging and biomedicine. *The journal of physical chemistry B* **110**, 7238-7248 (2006).

4 Qin, Z. & Bischof, J. C. Thermophysical and biological responses of gold nanoparticle laser heating. *Chemical Society Reviews* **41**, 1191-1217 (2012).

5 Wang, Y.-L. & Pelham Jr, R. J. in *Methods in enzymology* 298 489-496 (Elsevier, 1998).

6 Kandow, C. E., Georges, P. C., Janmey, P. A. & Beningo, K. A. Polyacrylamide hydrogels for cell mechanics: steps toward optimization and alternative uses. *Methods Cell Biol.* **83**, 29-46 (2007).

7 Arganda-Carreras, I. *et al.* Trainable Weka Segmentation: a machine learning tool for microscopy pixel classification. *Bioinformatics* **33**, 2424-2426 (2017).

8 Roca-Cusachs, P., Conte, V. & Trepat, X. Quantifying forces in cell biology. *Nat. Cell Biol.* **19**, 742 (2017).

9 Becerra, N. Y., López, B. L. & Restrepo, L. M. Thermosensitive behavior in cell culture media and cytocompatibility of a novel copolymer: poly (N-isopropylacrylamide-co-butylacrylate). *Journal of Materials Science: Materials in Medicine* **24**, 1043-1052 (2013).

10 Lowe, J. S., Chowdhry, B. Z., Parsonage, J. R. & Snowden, M. J. The preparation and physico-chemical properties of poly (N-ethylacrylamide) microgels. *Polymer* **39**, 1207-1212 (1998).

11 Zhang, Y., Furyk, S., Bergbreiter, D. E. & Cremer, P. S. Specific ion effects on the water solubility of macromolecules: PNIPAM and the Hofmeister series. *J. Am. Chem. Soc.* **127**, 14505-14510 (2005).

12 Park, T. G. & Hoffman, A. S. Sodium chloride-induced phase transition in nonionic poly(N-isopropylacrylamide) gel. *Macromolecules* **26**, 5045-5048 (1993).

13 Harmon, M. E., Jakob, T. A., Knoll, W. & Frank, C. W. A surface plasmon resonance study of volume phase transitions in N-isopropylacrylamide gel films. *Macromolecules* **35**, 5999-6004 (2002).

14 Halperin, A., Kröger, M. & Winnik, F. M. Poly (N-isopropylacrylamide) phase diagrams: fifty years of research. *Angewandte Chemie International Edition* **54**, 15342-15367 (2015).

15 Tanaka, T. & Fillmore, D. J. Kinetics of swelling of gels. *The Journal of Chemical Physics* **70**, 1214-1218 (1979).

16 Tanaka, T., Sato, E., Hirokawa, Y., Hirotsu, S. & Peetermans, J. Critical Kinetics of Volume Phase Transition of Gels. *Physical Review Letters* **55**, 2455-2458, doi:10.1103/PhysRevLett.55.2455 (1985).

17 Vogel, A. & Venugopalan, V. Mechanisms of Pulsed Laser Ablation of Biological Tissues. *Chem. Rev.* **103**, 577-644 (2003).

18 Song, A. S., Najjar, A. M. & Diller, K. R. Thermally Induced Apoptosis, Necrosis, and Heat Shock Protein Expression in Three-Dimensional Culture. *Journal of Biomechanical Engineering* **136**, 071006-1-071006-10 (2014).

19 Font, R. & Peria, F. The Finite Element Method with FreeFem++ for beginners. *Electronic Journal of Mathematics & Technology* **7** (2013).
20 Sunyer, R., Jin, A. J., Nossal, R. & Sackett, D. L. Fabrication of hydrogels with steep stiffness gradients for studying cell mechanical response. *PloS one* **7**, e46107 (2012).
21 Qian, H., Sheetz, M. P. & Elson, E. L. Single particle tracking. Analysis of diffusion and flow in two-dimensional systems. *Biophysical journal* **60**, 910-921 (1991).
22 Manzo, C. & Garcia-Parajo, M. F. A review of progress in single particle tracking: from methods to biophysical insights. *Reports on progress in physics* **78**, 124601 (2015).
