# Supplementary material for: Cellular responses to beating hydrogels to investigate mechanotransduction
Source: Nat Commun. 2019 Sep 6;10:4027. doi: 10.1038/s41467-019-11475-4 (PMC6731269; doi:10.1038/s41467-019-11475-4)
Supplement: Supplementary file 8 — Reporting Summary [file 41467_2019_11475_MOESM8_ESM.pdf]

nature research

Corresponding author(s): Prof. Dr.-Ing. Laura De Laporte

Last updated by author(s): Apr 18, 2019

Reporting Summary

Nature Research wishes to improve the reproducibility of the work that we publish. This form provides structure for consistency and transparency in reporting. For further information on Nature Research policies, see Authors & Referees and the Editorial Policy Checklist.

Statistics

For all statistical analyses, confirm that the following items are present in the figure legend, table legend, main text, or Methods section.

n/a	Confirmed	
☐	☒	The exact sample size (n) for each experimental group/condition, given as a discrete number and unit of measurement
☐	☒	A statement on whether measurements were taken from distinct samples or whether the same sample was measured repeatedly
☐	☒	The statistical test(s) used AND whether they are one- or two-sided<br>*Only common tests should be described solely by name; describe more complex techniques in the Methods section.*
☒	☐	A description of all covariates tested
☒	☐	A description of any assumptions or corrections, such as tests of normality and adjustment for multiple comparisons
☐	☒	A full description of the statistical parameters including central tendency (e.g. means) or other basic estimates (e.g. regression coefficient) AND variation (e.g. standard deviation) or associated estimates of uncertainty (e.g. confidence intervals)
☒	☐	For null hypothesis testing, the test statistic (e.g. F, t, r) with confidence intervals, effect sizes, degrees of freedom and P value noted<br>*Give P values as exact values whenever suitable.*
☒	☐	For Bayesian analysis, information on the choice of priors and Markov chain Monte Carlo settings
☒	☐	For hierarchical and complex designs, identification of the appropriate level for tests and full reporting of outcomes
☒	☐	Estimates of effect sizes (e.g. Cohen's d, Pearson's r), indicating how they were calculated

Our web collection on statistics for biologists contains articles on many of the points above.

Software and code

Policy information about availability of computer code

Data collection	Axiovision v 4.0, Las X, FLIR Research, Cell Tracker v1.0, Fiji v 1.52 b, Hokawo 2.10, DMA Q Serie 20.24.43. Nanoscope 9.40, Gen 5 2.0, Keyence VHM 174, WinTEM version V01.05.00.00, iTEM v5.2, UV spectra Manager version 2.15.04
Data analysis	Origin 2018 b, IBM SPSS Statistics 20, Adobe Illustrator cc 2018

For manuscripts utilizing custom algorithms or software that are central to the research but not yet described in published literature, software must be made available to editors/reviewers. We strongly encourage code deposition in a community repository (e.g. GitHub). See the Nature Research guidelines for submitting code & software for further information.

Data

Policy information about availability of data

All manuscripts must include a data availability statement. This statement should provide the following information, where applicable:

- Accession codes, unique identifiers, or web links for publicly available datasets
- A list of figures that have associated raw data
- A description of any restrictions on data availability

The authors declare that (the/all other) data supporting the findings of this study are available within the paper and its supplementary information files. Source data underlying Figures 1c,1e-f, 1h, 2g-j, 2l, 3a-c, 3h-j, 4e-j, 5b, 5d-e and Supplementary Figures 2b, 2d-f, 3a-f, 5c-e, 6, 7b-c, 8c-d, 9a-c, 10d, 11b-d, 12b-h, 14g, 15c, 24a-b, 26 are available upon request from the corresponding author.

Field-specific reporting

Please select the one below that is the best fit for your research. If you are not sure, read the appropriate sections before making your selection.

☒ Life sciences ☐ Behavioural & social sciences ☐ Ecological, evolutionary & environmental sciences

For a reference copy of the document with all sections, see nature.com/documents/nr-reporting-summary-flat.pdf

Life sciences study design

All studies must disclose on these points even when the disclosure is negative.

Sample size	Due to the exploratory nature of the study, sample size was the individual cells that were counted during analysis and the number of cells are mentioned in the figure caption.
Data exclusions	For image analysis, cells that do not fall in the field of view or divide were not included in the analysis
Replication	Multiple samples at various times were analyzed and experiments are performed at least 3 times.
Randomization	Not relevant
Blinding	Investigators were blinded during data analysis.

Reporting for specific materials, systems and methods

We require information from authors about some types of materials, experimental systems and methods used in many studies. Here, indicate whether each material, system or method listed is relevant to your study. If you are not sure if a list item applies to your research, read the appropriate section before selecting a response.

Materials & experimental systems

n/a	Involved in the study	
☐	☒	Antibodies
☐	☒	Eukaryotic cell lines
☒	☐	Palaeontology
☒	☐	Animals and other organisms
☒	☐	Human research participants
☒	☐	Clinical data

Methods

n/a	Involved in the study	
☒	☐	ChIP-seq
☒	☐	Flow cytometry
☒	☐	MRI-based neuroimaging

Antibodies

Antibodies used	anti MRTFA primary Antibody - Santa Cruz biotech SC 398675 LOT A2517<br>YAP Primary Antibody - Cell signaling Technologies 4912S Lot 5<br>anti Fibronectin Antibody - Sigma F 3648 lot # 018M4752V<br>anti paxillin Antibody - Sigma SAB 4502553<br>anti vinculin Antibody - Sigma V 9264<br>Alexa Fluor 488 anti-rabbit secondary A 11034 LOT 1885241<br>Alexa Fluor 633 anti-mouse secondary A 21050 LOT 1845042<br>Alexa Fluor 633 anti rabbit secondary A21071 LOT 1932492<br>Alexa Fluor 488 anti-mouse secondary A11001 LOT 1834337<br>cytopainter i - Fluor 594 Abcam ab 176757 LOT GR 3214552-17
Validation	Protocols for immunofluorescence were optimized (antibody dilution and incubation time), the application of each antibody for the species is noted as per the manufacturers protocol.<br>All antibodies used were well described in literature or in the manufacturers' protocols. No novel antibodies are used in this study.

Eukaryotic cell lines

Policy information about cell lines

Cell line source(s)	L 929
Authentication	Commercially available cell line is purchased from the Deutsche Sammlung von Mikroorganismen und Zellenkulturen GmbH

Authentication	(DSMZ) No ACC-2
Mycoplasma contamination	Mycoplasma contamination was not observed after dapi staining.
Commonly misidentified lines<br>(See ICLAC register)	No commonly misidentified cell lines were used.
